# A new mechanism for reduced sensitivity to demethylation‐inhibitor fungicides in the fungal banana black Sigatoka pathogen *Pseudocercospora fijiensis*

**DOI:** 10.1111/mpp.12637

**Published:** 2018-02-13

**Authors:** Caucasella Diaz‐Trujillo, Pablo Chong, Ioannis Stergiopoulos, Viviane Cordovez, Mauricio Guzman, Pierre J. G. M. De Wit, Harold J. G. Meijer, Gabriel Scalliet, Helge Sierotzki, Esther Lilia Peralta, Rafael E. Arango Isaza, Gerrit H. J. Kema

**Affiliations:** ^1^ Wageningen University and Research, Wageningen Plant Research 6700 AA Wageningen the Netherlands; ^2^ Wageningen University and Research Laboratory for Phytopathology 6700 AA Wageningen the Netherlands; ^3^ Escuela Superior Politécnica del Litoral, ESPOL, Centro de Investigaciones Biotecnológicas del Ecuador, CIBE, Laboratorio de Fitopatología ESPOL Polythecnic University Guayaquil 09‐01‐5663 Ecuador; ^4^ Department of Plant Pathology University of California, Davis Davis CA 95616‐8751 USA; ^5^ Department of Microbial Ecology Netherlands Institute of Ecology Wageningen 6708 PB the Netherlands; ^6^ Department of Phytopathology, National Banana Corporation of Costa Rica (CORBANA), La Rita de Pococí Limón 6504‐1000 Costa Rica; ^7^ Crop Protection Disease Control, Syngenta Crop Protection Münchwilen AG Stein 4333 Switzerland; ^8^ Plant Biotechnology Unit Corporación para Investigaciones Biológicas (CIB) Medellín 050034 Colombia; ^9^ School of Biosciences, Faculty of Sciences National University of Colombia Medellín 050034 Colombia

**Keywords:** DMI, fungicide, *Pfcyp51* promoter

## Abstract

The Dothideomycete *Pseudocercospora fijiensis*, previously *Mycosphaerella fijiensis*, is the causal agent of black Sigatoka, one of the most destructive diseases of bananas and plantains. Disease management depends on fungicide applications, with a major contribution from sterol demethylation‐inhibitors (DMIs). The continued use of DMIs places considerable selection pressure on natural *P. fijiensis* populations, enabling the selection of novel genotypes with reduced sensitivity. The hitherto explanatory mechanism for this reduced sensitivity was the presence of non‐synonymous point mutations in the target gene *Pfcyp51*, encoding the sterol 14α‐demethylase enzyme. Here, we demonstrate a second mechanism involved in DMI sensitivity of *P. fijiensis*. We identified a 19‐bp element in the wild‐type (wt) *Pfcyp51* promoter that concatenates in strains with reduced DMI sensitivity. A polymerase chain reaction (PCR) assay identified up to six *Pfcyp51* promoter repeats in four field populations of *P. fijiensis* in Costa Rica. We used transformation experiments to swap the wt promoter of a sensitive field isolate with a promoter from a strain with reduced DMI sensitivity that comprised multiple insertions. Comparative *in vivo* phenotyping showed a functional and proportional up‐regulation of *Pfcyp51*, which consequently decreased DMI sensitivity. Our data demonstrate that point mutations in the *Pfcyp51* coding domain, as well as promoter inserts, contribute to the reduced DMI sensitivity of *P. fijiensis*. These results provide new insights into the importance of the appropriate use of DMIs and the need for the discovery of new molecules for black Sigatoka management.

## Introduction

Black Sigatoka, caused by the ascomycete *Pseudocercospora fijiensis* (Morelet, 1969) Deighton (1976) [previously *Mycosphaerella fijiensis* Morelet (1969)], is one of the most devastating and economically significant diseases of export bananas and plantains. Disease management is mainly based on the extensive application of primarily single‐site fungicides. However, the continuous sexual reproduction of *P. fijiensis* generates genetically highly diverse and hence versatile populations that quickly adapt to changing environments, including extensive fungicide treatments (Arango Isaza *et al*., 2016; Conde‐Ferráez *et al*., 2007; Hayden and Carlier, 2003; Rivas *et al*., 2004; Romero and Sutton, 1997). As a result, reduced fungicide efficacy develops frequently and spreads rapidly (Arango Isaza *et al*., 2016). This situation has contributed to a grave increase in the number of fungicide applications, which can reach over 50 applications per year (maximum of 10 applications with sterol 14α‐demethylation‐inhibitors, DMIs) in most banana export countries (Chong‐Aguirre, 2016; De Lapeyre De Bellaire *et al*., 2010; FRAC, 2010; Martínez‐Bolaños *et al*., 2012), thereby frequently comprising a 30% share of the production costs (Marín *et al*., 2003). This practice poses a threat to the occupational health of plantation workers and the environment if guidelines are not followed. It is thus imperative to understand the mechanisms by which reduced fungicide efficacy develops to enable adequate long‐term disease management strategies with optimized chemical input.

Azole fungicide applications against black Sigatoka started in 1987 and have been widely used since 1991 when propiconazole, one of the major contemporary DMIs, was introduced to the market (Chong‐Aguirre, 2016; Romero and Sutton, 1997). Currently, several DMIs, such as difenoconazole, bitertanol and epoxiconazole, are used in disease management programmes, either alone or in mixtures with other fungicides with different modes of action. DMIs inhibit the activity of the CYP51 enzyme which is involved in the 14α‐demethylation of the ergosterol precursor eburicol (24‐methylene‐24,25‐dihydrolanosterol). Ergosterol regulates cellular membrane fluidity and permeability and is essential for cell viability (Lepesheva and Waterman, 2011). However, reduced efficacy of single‐site fungicides surfaced rapidly in *P. fijiensis* after the introduction of quinone outside inhibitors (QoIs or strobilurins), methyl benzimidazole carbamates (MBCs) and DMIs for disease control in banana production (Amil *et al*., 2007; Arango Isaza *et al*., 2016; Cañas‐Gutiérrez *et al*., 2006, 2009; Romero and Sutton, 1997). Previous studies on *P. fijiensis* have revealed the correlation between the reduced efficacy of propiconazole and point mutations in the coding domain of the *Pfcyp51* gene, which cause non‐synonymous amino acid substitutions surrounding the substrate recognition sites (SRSs) at positions Y136, A313, Y461 and Y463 (Cañas‐Gutiérrez *et al*., 2009; Chong‐Aguirre, 2016). Until now, this was the only explanatory mechanism for reduced sensitivity towards azoles in *P. fijiensis*. Here, we introduce an additional mechanism that drives reduced sensitivity to DMIs in *P. fijiensis*. We identified the presence of one or more repetitive elements in the promoter region of *Pfcyp51* amongst *P. fijiensis* field isolates with reduced DMI sensitivity, and catalogued such variants in 225 field isolates originating from various (treated and untreated) banana plantations in Costa Rica. Comparison with 14 control isolates from Ecuador, Asia and Africa showed a positive correlation between the presence and copy number of the *Pfcyp51* promoter elements, *Pfcyp51* overexpression and reduced DMI sensitivity. We subsequently established the functional relationship between the number of promoter inserts, increased target expression and reduced DMI sensitivity through *Pfcyp51* promoter swapping experiments between wild‐type (wt) isolates and *P. fijiensis* strains with reduced DMI sensitivity. We thereby formally demonstrated a novel mechanism involved in reduced fungicide efficacy of DMIs to *P. fijiensis*, in addition to the described target site mutations in the coding sequence of *Pfcyp51*.

## Results

### *In vitro* sensitivity to propiconazole

The *P. fijiensis* isolates that were tested for sensitivity to propiconazole were classified into three groups: (1) sensitive isolates with 50% inhibitory concentration (EC_50_) ≤ 0.10 mg/L; (2) moderately resistant isolates with EC_50_ = 0.10–1.0 mg/L; and (3) resistant isolates with EC_50_ > 1.0 mg/L (Table 1). Among the 25 isolates tested for sensitivity to propiconazole, seven were sensitive, 14 were moderately resistant and four were resistant. Clear cross‐resistance between propiconazole and cyproconazole was observed, as the majority of isolates showed similar EC_50_ values (Table 1; Fig. S1, see Supporting Information).

**Table 1:** Origin and characteristics of the *Pfcyp51* gene and its promoter in 25 *Pseudocercospora fijiensis* isolates used in this study, including their sensitivity to propiconazole and cyproconazole (half‐maximal effective concentration, EC_50_).

																Propiconazole		Cyproconazole	
Origin	Isolate	Promoter insertion	Repetitive units	CYP51 modulations												EC_50_ (mg/L)	SD	EC_50_ (mg/L)	SD
Burundi	X849	wt	1			V106D										0.004	0.002	0.006	0.001
Cameroon	C_86	wt	1													<0.001^*^	–	<0.001^*^	–
Ecuador	RS_13	wt	1	T18I		V106D				A313G					Y463N	0.112	0.052	0.121	0.060
Ecuador	E_22	wt	1	T18I		V106D										0.011	0.009	<0.001^*^	
Ecuador	GS_10	wt	1	T18I		V106D				A313G					Y463N	0.291	0.050	0.552	0.208
Ecuador	GS_4	wt	1	T18I		V106D				A313G					Y463N	0.481	0.047	0.666	0.253
Ecuador	RN_3	wt	1	T18I		V106D				A313G					Y463H	0.284	0.011	0.514	0.059
Ecuador	RN_5	wt	1	T18I		V106D				A313G					Y463H	0.420	0.285	0.843	0.242
Ecuador	SaR_2	wt	1	T18I		V106D				A313G			Y461D			0.197	0.085	0.231	0.067
Ecuador	SaR_5	wt	1	T18I		V106D				A313G					Y463N	0.214	0.121	0.611	0.284
Gabon	X851	wt	1			V106D										<0.001^*^	–	0.010	0.002
Indonesia	X845	wt	1	T18I	Y58F	V106D										0.009	0.011	0.003	0.001
Philippines	X846	wt	1	T18I		V106D	V116L									0.007	0.006	0.002	0.001
Taiwan	X847	wt	1			V106D			K171R			A446S				<0.001^*^	–	<0.001^*^	–
Costa Rica	Z4_14	wt	1	T18I		V106D				A313G					Y463D	0.214	0.005	0.674	0.294
Costa Rica	Z8_17	wt	1	T18I		V106D				A313G					Y463S	0.158	0.092	0.633	0.264
Costa Rica	Z4_16	wt	1	T18I		V106D					A381G			G462A		0.166	0.047	0.521	0.080
Costa Rica	Z4_7	wt	1	T18I		V106D				A313G					Y463S	0.266	0.115	0.489	0.153
Costa Rica	Z4_11	wt	1	T18I		V106D				A313G					Y463H	0.112	0.033	0.561	0.225
Costa Rica	Ca1_5	CTCGTACGATAGCACAAATGTTAAATCTCGTACGATAGC	3	T18I		V106D		Y136F			A381G				Y463D	1.144	0.209	1.883	1.055
Costa Rica	Z8_12	CTCGTACGATAGCACAAATGTTAAATCTCGTACGATAGC	3	T18I		V106D		Y136F							Y463D	0.188	0.040	0.403	0.146
Costa Rica	Z8_18	CTCGTACGATAGCACAAATGTTAAATCTCGTACGATAGC	3	T18I		V106D		Y136F							Y463D	0.153	0.056	0.784	0.514
Costa Rica	Ca10_13	AAATCTCGTACGATAGCATAAAATCTCGTACGATAGCATAAAATCTCGTACGATGTTAAATCTCGTACGATAGCATAAATCTCGTACGATAGCACCTGCC	6	T18I		V106D		Y136F	–						Y463D	3.346	0.725	>10^*^	–
Costa Rica	Ca5_16	AAATCTCGTACGATAGCATAAAATCTCGTACGATAGCATAAAATCTCGTACGATGTTAAATCTCGTACGATAGCATAAATCTCGTACGATAGCACCTGCC	6	T18I		V106D		Y136F							Y463D	2.292	0.420	>10^*^	–
Costa Rica	Ca6_11	AAATCTCGTACGATAGCATAAAATCTCGTACGATAGCATAAAATCTCGTACGATGTTAAATCTCGTACGATAGCATAAATCTCGTACGATAGCACCTGCC	6	T18I		V106D		Y136F							Y463D	2.75	0.13	7.979	3.293

^*^Out of dose range for calculations.

SD, standard deviation; wt, wild‐type.

### *Pseudocercospora fijiensis* isolates with reduced sensitivity always contain repetitive elements in the *Pfcyp51* promoter

**Figure 1:**
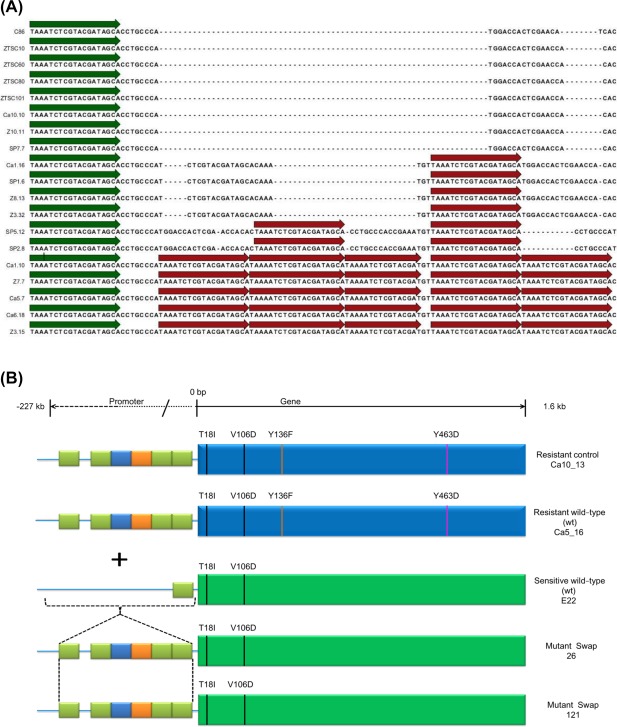
The *Pfcyp51* structure. (A) Alignment of the promoter regions of the *Pfcyp51* gene of *Pseudocercospora fijiensis* isolates collected from the Zent (Z), Cartagena (Ca), San Pablo (SP) and the wild‐type (wt) San Carlos (ZTSC) banana plantations in Costa Rica; isolate CIRAD86 (C86) is the reference wt isolate; the repeat element present in all isolates at position −122 bp is shown by the green arrows and additional repeated elements identified in various *P. fijiensis* isolates are shown as red arrows (see Table 1 for origin of isolates). (B) Configuration of the *Pfcyp51* promoter and coding domains of the wt *P. fijiensis* isolates used to generate transformants. The promoter region is shown on the left as a blue line with different coloured boxes; green, blue and orange boxes represent the 19‐bp, 20‐bp and 16‐bp promoter repeat elements, respectively; rectangular boxes on the right represent the coding regions of the *Pfcyp51* gene in these isolates: green represents the sensitive wt and blue denotes the resistant donor (resistant wt) coding region. Vertical lines in the coding regions represent amino acid substitutions.

**Figure 2:**
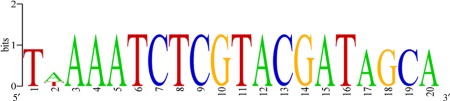
Sequence logo of the *Pfcyp51* promoter repeat element. Sequences of all repeat elements were aligned and used to generate the consensus sequence. The logo displays the frequency of the nucleotides within the repeated elements of 16, 19 or 20 bp that were observed in the promoter of *Pfcyp51*.

Detailed comparison between the *Pfcyp51* promoter sequences from resistant isolates and the reference *P. fijiensis* isolate CIRAD86 revealed that resistant isolates possessed an insertion in the promoter at 103 bp upstream from the start codon. Meanwhile, some isolates with reduced sensitivity showed a shorter insertion than resistant strains at the same position. Likewise, sensitive isolates did not show any insertion. Insertions comprised repeats of 19‐bp elements ‘TAAATCTCGTACGATAGCA’ present once in the *Pfcyp51* promoter at 122 bp upstream from the start codon, at scaffold 7:2121794–2121813 of the CIRAD86 reference (*Pseudocercospora fijiensis* v2.0, Joint Genome Institute) (Figs 1 and 2).

Some isolates contained part of the element in their insertions, whereas others had a modified element with a few additional nucleotides. In addition to the 19‐bp element, slightly modified 16‐bp (TAAAATCTCGTACGAT) and 20‐bp (TAAAATCTCGTACGATAGCA) elements were also present in the *Pfcyp51* promoter. For example, in resistant isolates Ca1_5, Ca5_16, Ca6_11 and Ca10_13 (Table 1; Text S1, see Supporting Information), the basic 19‐bp element was repeated up to six times (four fully conserved and one partial, mostly in tandem insertion) and thrice in the moderately resistant *P. fijiensis* isolates Z8_12 and Z8_18. DNA sequence analysis of the resistant isolates from Costa Rica (Ca5_16, Ca6_11 and Ca10_13) revealed that these contained identical mutations in the coding region of the *Pfcyp51* gene, and that the overall length of the *Pfcyp51* promoter inserts accumulated to 100 bp (Table 1).

### Repetitive elements in the promoter of *Pfcyp51* up‐regulate its expression

**Figure 3:**
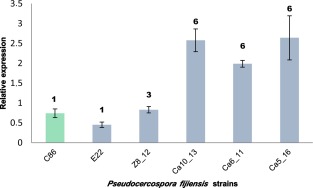
Relative expression of *Pfcyp51* (normalized to the *Pseudocercospora fijiensis* actin gene) in six *P. fijiensis* isolates carrying different numbers of promoter inserts (indicated on the top of each bar). Reference isolate CIRAD86 (C86) is shown in green. Data represent the averages of three biological repetitions each with at least three technical replicates (error bars indicate standard deviations).

To test whether *Pfcyp51* gene expression is affected by the presence of repetitive elements, we quantified the expression in mycelium by real‐time reverse transcription‐polymerase chain reaction (RT‐PCR), normalized to the expression of the actin gene (*Pfact*), relative to wt controls. *Pseudocercospora fijiensis* isolates Ca5_16, Ca6_11 and Ca10_13, all containing six repeat elements in the *Pfcyp51* promoter, showed a 3.3–5.6‐fold increase in *Pfcyp51* gene expression relative to control isolate E22, and a smaller difference from the other control strain CIRAD86 that only contained the basic 19‐bp element (Fig. 3). In contrast, no significant difference was found between the control isolate CIRAD86 and *P. fijiensis* isolate Z8_12, which has three repeat elements. The up‐regulation of *Pfcyp51* was constitutive and independent of the addition of propiconazole to the culture medium (data not shown).

### *Pfcyp51* promoter insertions accumulate in *P. fijiensis* strains with reduced fungicide sensitivity originating from frequently sprayed commercial banana plantations in Costa Rica

**Figure 4:**
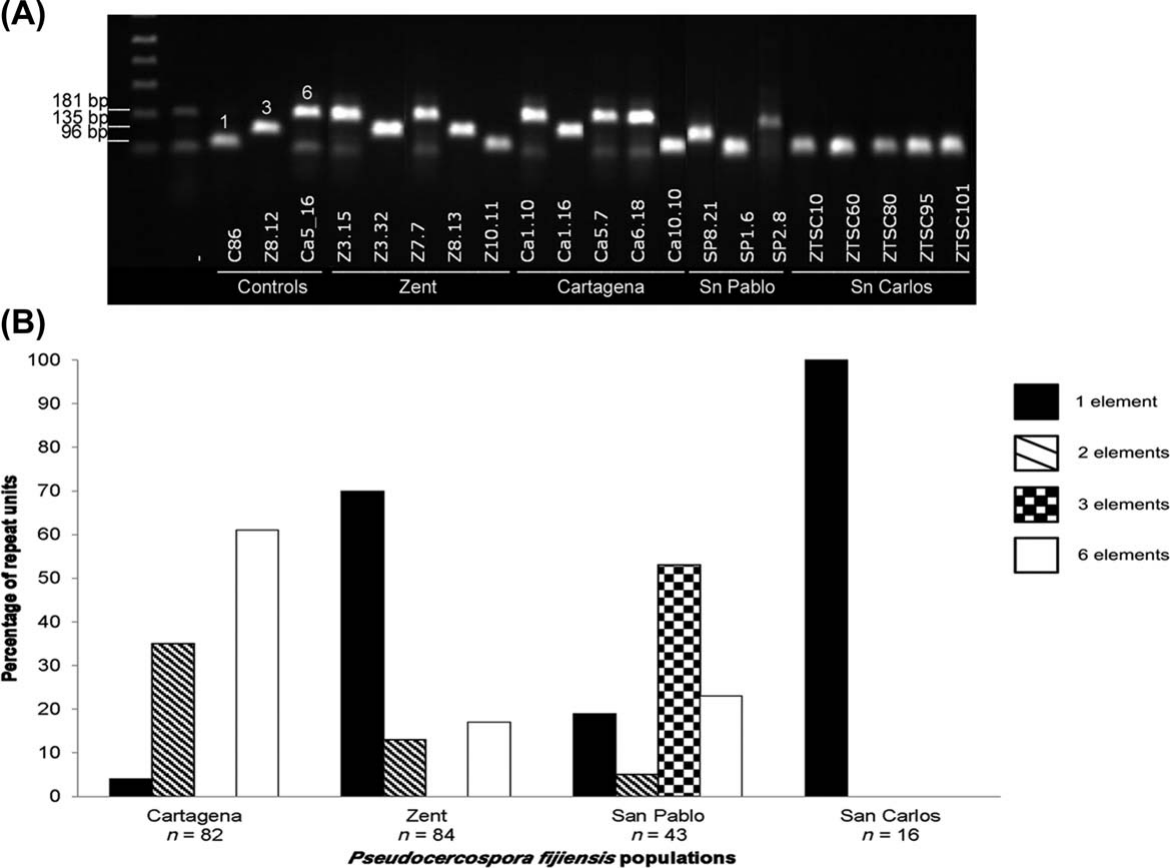
Quantification of the number of *Pfcyp51* promoter repeats in *Pseudocercospora fijiensis* isolates from four banana plantations in Costa Rica. (A) Example of polymerase chain reaction (PCR) amplification of the *Pfcyp51* promoter in isolates from different populations. Isolate CIRAD86 (C86) was used as a control for the presence of one repeat element, Z8.12 as a control with three repeat elements and Ca5_16 as a control with six repeat elements. The number of repeat elements in each control sample is indicated above the corresponding amplicon. The other isolates originated from banana plantations extensively treated (or not) with azole fungicides and contain varying numbers of repeat elements in the *Pfcyp51* promoter. (B) Distribution of repeat elements in the *Pfcyp51* promoter within Costa Rican populations of *P. fijiensis*, based on 225 PCR amplifications.

To identify the number of repeat element copies in the *Pfcyp51* promoter, we performed PCR analyses on 225 isolates originating from four banana plantations in Costa Rica that have been studied previously (Arango Isaza *et al*., 2016): three plantations (Cartagena, Zent and San Pablo) with intensive fungicide applications and one unsprayed plantation (ZTSC or San Carlos). Comparison of the amplicon sizes by gel electrophoresis and sequence data revealed banding patterns that corresponded to two, three and six promoter repeats (Fig. 4).

Isolates containing six repeat elements dominated (50 of 82) the Cartagena population, followed by isolates with two copies (29 of 82), whereas isolates with only the original 19‐bp element were scarce (three of 82). In contrast, the Zent population was dominated by isolates with only the 19‐bp element in the *Pfcyp51* promoter (59 of 84), but isolates containing two and six promoter repeats were also found (11 and 14 of 84, respectively). The San Pablo population was dominated by a genotype with three promoter repeats (23 of 43), which was not observed in the other populations, in addition to strains with one (eight of 23) and two (two of 23) promoter repeats. None of the genotypes with accumulated promoter repeats were observed in the San Carlos populations that exclusively comprised *P. fijiensis* strains with the original 19‐bp element in the *Pfcyp51* promoter (Fig. 4).

Sequence analyses revealed that the accumulated promoter repeat elements varied from 42 bp (two elements), 59 bp (three elements) up to 100 bp (six elements). All repeat elements were inserted exactly 103 bp upstream of the start codon of *Pfcyp51* and were 20 bp (TAAAATCTCGTACGATAGCA), 19 bp (TAAATCTCGTACGATAGCA) or 16 bp (TAAAATCTCGTACGAT) in length and concatenated in tandem, or were separated by a few nucleotides. Elements of 20 and 19 bp only differ by one extra adenine, whereas the 16‐bp element represents a shorter version of the 19‐bp insert (Fig. 1). The 19‐bp element was found in isolates with one, two and three copies, whereas, in isolates with six *Pfcyp51* promoter inserts, the 19‐bp element was always accompanied by single inserts of the 16‐ and 20‐bp units. Hence, the 19‐bp element was the most common insertion across all isolates analysed (Fig. 1).

### Analysis of the *Pfcyp51* coding sequence

As expected, sequence analyses of different isolates revealed the presence of non‐synonymous mutations in the coding region of *Pfcyp51*. These resulted in amino acid changes Y136F, A313G and Y463D/H/N which have been reported previously and associated with reduced sensitivity to propiconazole (Cañas‐Gutiérrez *et al*., 2009). Here, we identified nine new amino acid substitutions (T18I, Y58F, V106D, V116L, K171R, A381G, A446S, G462A and Y463S) (Table 1). All isolates contained the T18I and V106D substitutions. Apart from these, the most frequent amino acid substitutions, A313G and Y463N/D/S/H, were observed in 11 and 16 of 25 isolates, respectively. These mutations were often found in combination with Y136F and A381G. Thus, the most frequently observed haplotypes amongst the 25 isolates were T18I, V106D, Y136F, A313G and Y463D/N/S, which were found in combination with two, three or six copies of the *Pfcyp51* repeat element. Strains with the T18I, V106D, Y136F and Y463D *Pfcyp51* modifications showed the least sensitivity to the tested fungicides. In addition, several other combinations of amino acid substitutions were observed in the analysed cohort of *P. fijiensis* isolates, including A313G and Y463S/H/D/N; A381G and G462A; Y136F and Y463D; Y136F, A381G and Y463D; and K171R and A446S.

### Functional analysis of the *Pfcyp51* promoter insertions

**Figure 5:**
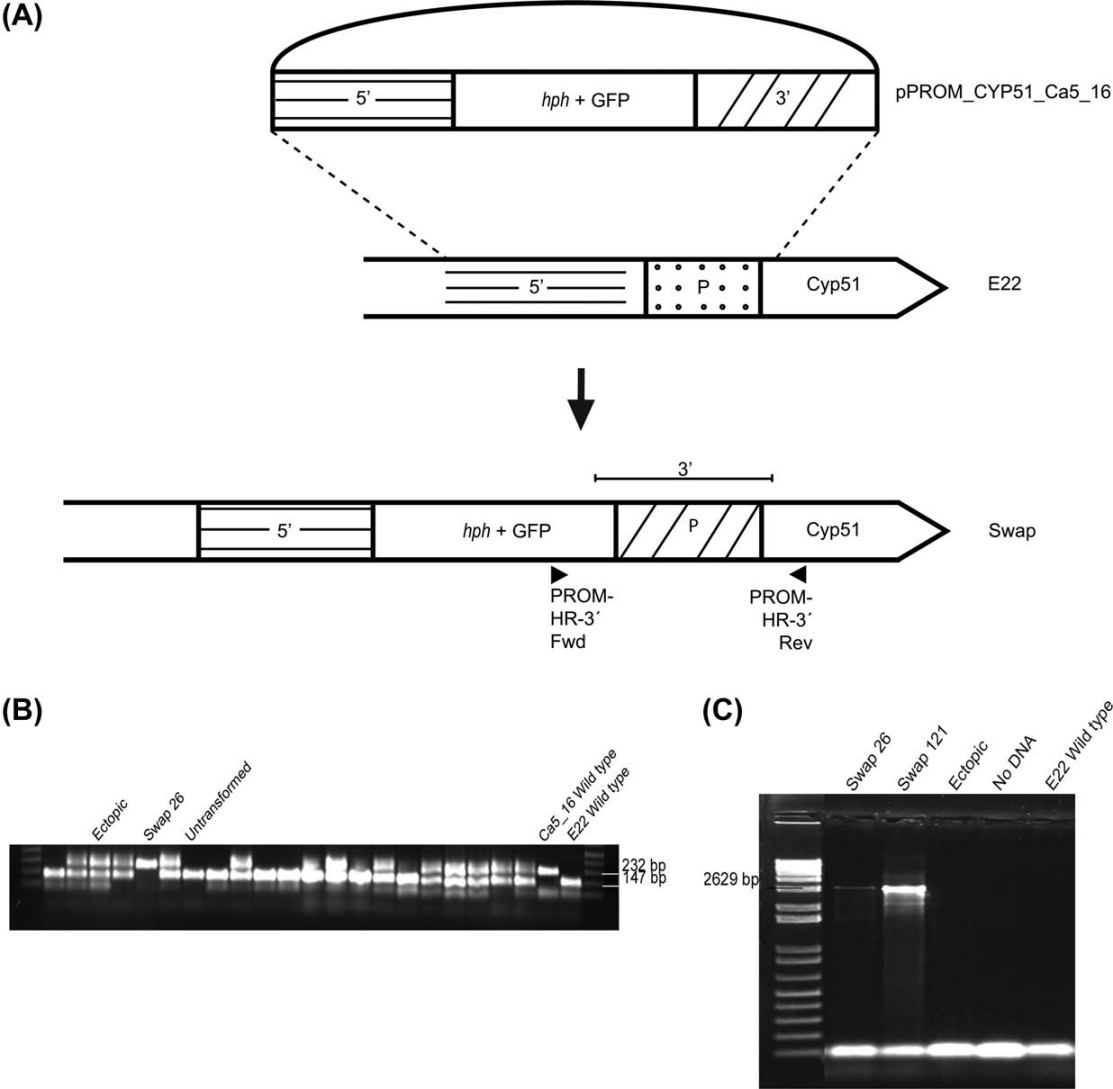
Transformation design to swap *Pfcyp51* promoters of *Pseudocercospora fijiensis* isolates. (A) Isolate Ca5_16 is the *Pfcyp51* promoter donor with six repeat elements (slashed area part with the cross lines). The 3′ and 5′ recombination fragments (crossed out area part with the horizontal lines) were amplified with CYP‐Prom primers and ligated to a cassette with the hph and green fluorescent protein (GFP) markers into construct pPROM_CYP51_Ca5_16. The *P. fijiensis* E22 sensitive isolate with one 19‐bp promoter element (dotted area) was transformed with this construct. (B) The promoter lengths of positive GFP‐tagged transformants were amplified and compared with the donor and wild‐type (wt) recipient isolate. Transformant Swap 26 is shown as an example of a promoter replacement transformant, with a similar amplicon to the donor isolate. Ectopic transformants possess the promoter fragment of both the donor and the recipient isolate, whereas untransformed isolates only show the wt‐sized amplicon. (C) Detection and characterization of promoter swapped transformants were performed by amplification of the 2629‐bp cassette between the homologous recombination sites and the *Pfcyp51* coding region using primers PROM‐HR‐3′ on GFP fluorescent transformants with a promoter amplicon similar to the donor isolate.

We discovered a range of promoter insertions in *P. fijiensis* isolates from banana plantations that had been treated with fungicides. These promoter insertions, in particular the six repeat inserts, conferred enhanced expression of *Pfcyp51*. The isolates carrying these insertions also displayed reduced sensitivity to DMI fungicides, but also carried a *Pfcyp51* mutation in the coding sequence, which was hitherto the only explanatory mechanism for reduced DMI sensitivity. To disentangle the relationship between mutations in the coding sequence and the promoter insertions, we introduced the *Pfcyp51* promoter from the resistant *P. fijiensis* isolate Ca5_16 with six repeat elements into the sensitive wt E22 isolate from Ecuador (Table 1; Fig. 5).

**Figure 6:**
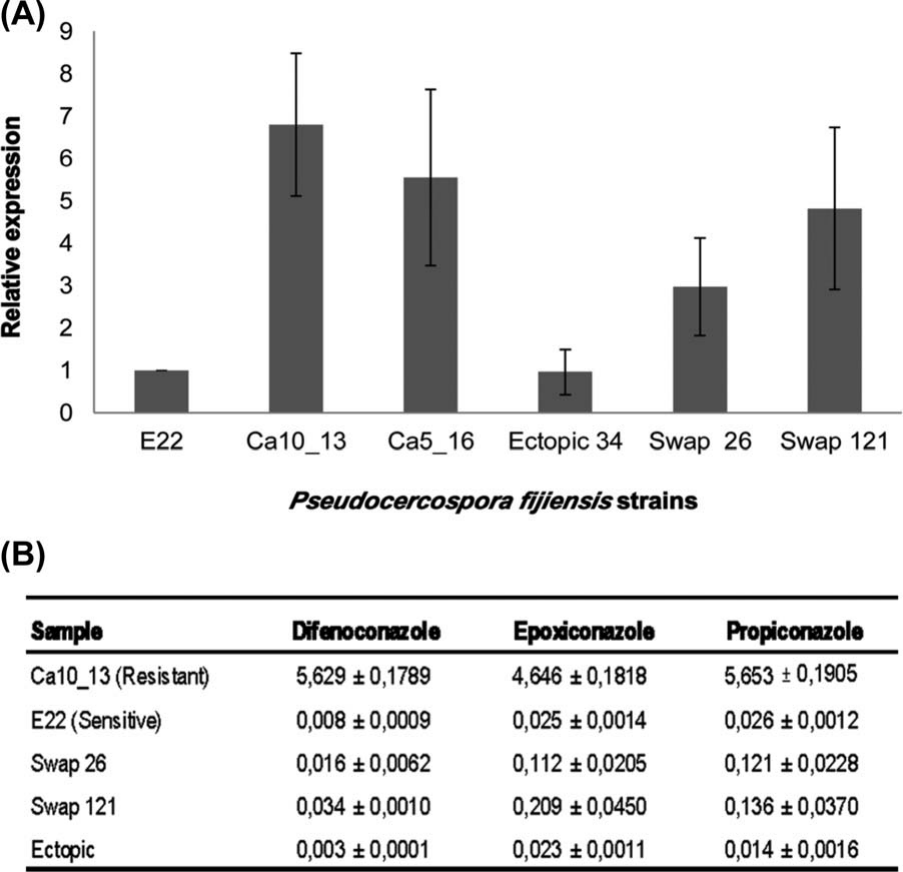
*In vitro* sensitivity of *Pseudocercospora fijiensis* transformants Swap 26 and Swap 121 with swapped *Pfcyp51* promoters vs. various control isolates. (A) The relative expression [normalized to the expression in wild‐type (wt) sensitive donor isolate E_22) of *Pfcyp51* in Swap 26 and Swap 121, the wt E22 and the resistant isolate (Ca10_13) with identical promoter and coding region to the donor isolate (Ca5_16), as well as the ectopic control isolate (Ectopic 34). Data represent the averages of three replications. (B) Table with means of the 50% inhibitory concentration (EC_50_) (mg/L) of the *P. fijiensis* promoter swapped transformants Swap 26 and Swap 121 and various control isolates to three azole fungicides.

Transformation of wt *P. fijiensis* isolate E22 resulted in 250 green fluorescent protein (GFP)‐ and hygromycin (*hph*)‐positive transformants. The transformants were characterized by PCR to differentiate isolates with six repeats in the *Pfcyp51* promoter at the correct integration site from ectopic transformants (Fig. 5). Two independent transformants, Swap 26 and Swap 121, showing the Ca5_16 promoter amplicon and positive for the correct integration site, were selected for further analyses (Fig. 5). Subsequently, we performed qRT‐PCR analyses on Swap 26 and Swap 121, together with the *P. fijiensis* control isolates comprising the recipient wt isolate E22 and the wt resistant isolates Ca5_16 and Ca10_13 and an ectopic transformant. Consistent with previous results, the resistant isolates Ca5_16 and Ca10_13 expressed *Pfcyp51* at a higher level than did the wt E22 recipient isolate. Moreover, the expression of *Pfcyp51* was significantly increased in both Swap 26 and Swap 121 compared with wt strain E22 and the ectopic isolate, and not significantly different from the resistant donor isolate Ca5_16 (Fig. 6). Hence, these results demonstrate that replacement of the *Pfcyp51* promoter from a sensitive *P. fijiensis* isolate by the promoter from a resistant strain results in the overexpression of *Pfcyp51*.

To determine whether the observed effect was independent of azole fungicides, we challenged the transformants with difenoconazole, epoxiconazole and propiconazole in 96‐well plates and calculated the EC_50_ values. A consistent growth pattern was observed for all controls (0 mg/L). The wt strain Ca10_13 grew up to 2.56 mg/L of difenoconazole or epoxiconazole, and 10.24 mg/L of propiconazole (wt isolate Ca5_16 was removed because of contamination). The sensitive wt isolate E22 and the ectopic transformant only grew up to 0.016 mg/L of difenoconazole and 0.04 mg/L of epoxiconazole or propiconazole. The Swap 26 and Swap 121 transformants grew on DMI concentrations that were at least fourfold higher than those of the sensitive wt control isolate E22. For difenoconazole, transformants Swap 26 and Swap 121 displayed a twofold and over fourfold (4.25) increase in EC_50_ compared with the sensitive wt isolate E22, respectively (Fig. 6). For epoxiconazole, Swap 26 displayed a 4.48‐fold reduction in sensitivity, whereas Swap 121 displayed a slightly higher 8.36‐fold reduction. Finally, EC_50_ for propiconazole of the wt strain E22 was 4.65‐fold and 5.23‐fold lower than that of Swap 26 and Swap 121, respectively. The ectopic transformant displayed a similar sensitivity to wt E22 regardless of the fungicide used (Fig. 6). These data confirm that *Pfcyp51* promoter modifications contribute to reduced DMI efficacy in *P. fijiensis*.

## Discussion

Disease management in agricultural crops is commonly based on an integrated approach comprising host resistance, agronomic measures and crop protection agents whenever necessary (Matthews *et al*., 2014). As a result of the ubiquity of ‘Cavendish’ clones, which represent over 90% of the global banana trade, and their vulnerability to *P. fijiensis*, disease control in banana almost entirely relies on crop protection agents and prophylactic measures. Despite the use of decision support systems accompanied by leaf surgery and the removal of infected foliage to reduce the inoculum potential, the cornerstone for *P. fijiensis* control remains chemical crop protection, with the emphasis on azole fungicides (Price *et al*., 2015). Consequently, the selection pressure on the pathogen has been enormous and has resulted in the appearance of *P. fijiensis* populations with reduced fungicide sensitivity, which calls for a better understanding of its origin and dissemination.

The presence of mutations in the *Pfcyp51* gene has been related previously to propiconazole resistance in *P. fijiensis* (Cañas‐Gutiérrez *et al*., 2009). Here, we have focused on the promoter region as an important determinant for *Pfcyp51* gene expression, and describe the identification of a 19‐bp element, whose concatenation up‐regulates *Pfcyp51* expression and confers reduced DMI sensitivity. Our data represent the first report of the targeted genetic modification of *P. fijiensis* to demonstrate a new mechanism for DMI sensitivity modulation in this organism.

PfCYP51 substitutions Y136F, A313G, A381G, Y461D, Y463D, Y463H and Y463N were found in the present study, in accordance with previous observations for *P. fijiensis* exposed to propiconazole (Cañas‐Gutiérrez *et al*., 2009), as well as in exposure to other azoles in *Zymoseptoria tritici*, *Candida albicans*, *Pyrenophora teres* f. sp*. teres* and *Aspergillus fumigatus* (Akins and Sobel, 2009; Cools and Fraaije, 2013; Mair *et al*., 2016; Mellado *et al*., 2007). Unexpectedly, we identified a 100‐bp insertion in the *Pfcyp51* promoter region in addition to the coding region mutations in most *P. fijiensis* isolates from the Cartagena population. These insertions comprise six copies of a repetitive element, whereas a single copy of this element is present in all sensitive isolates. Isolates with reduced sensitivity usually have two, three or more copies of this element (Chong‐Aguirre, 2016).

Unlike in *P. teres* f. sp. *teres* (Mair *et al*., 2016) and *Erysiphe necator* (Rallos and Baudoin, 2016), which showed overexpression of *Cyp51*, but no promoter modification, changes in the promoter region of the *cyp51* gene have been described in other fungi. Such changes comprise repeated promoter elements, truncated derivatives of a LINE‐like retrotransposon in *Blumeriella jaappi* (Ma *et al*., 2006), a MITE‐like transposon named PdMLE1 in *Penicillium digitatum* (Sun *et al*., 2013), a larger transposon of 1.8 kb in *A. fumigatus* (Albarrag *et al*., 2011; Verweij *et al*., 2013) or transcription factor binding sites in *Venturia inaequalis* (Villani *et al*., 2016). More detailed studies are required in *P. fijiensis* to determine whether the repeat elements observed here correspond to the movement of a transposon sequence, or whether *Pfcyp51* expression is possibly co‐regulated by transposons. However, unlike previous reports of promoter insertions with 199 bp to 5.6 kbp sequence transposons in *V. inaequalis* (Schnabel and Jones, 2001; Villani *et al*., 2016), the *Pfcyp51* promoter insertion merely comprises 19‐bp elements, or minor 16‐bp and 20‐bp variants, which accumulate up to 100 bp in length, shorter than the insertions in *V. inaequalis* and *Z. tritici* (Cools *et al*., 2012), where no transposons were reported. The insertions in the *Pfcyp51* promoter are shorter than any promoter insertions reported in *A. fumigatus* (Snelders *et al*., 2012; Verweij *et al*., 2007) and *Pyrenopeziza brassicae* (Carter *et al*., 2014). In other organisms, e.g. *Escherichia coli*, overexpression of a desired gene was achieved by tandem repeats of core promoter sequences called ‘MCPtacs’ (Li *et al*., 2012). In this way, a larger number of mutations in the coding region could be controlled, which would compromise the activity of the enzyme and hence reduce sensitivity (Cools *et al*., 2012; Leroux and Walker, 2011). Possibly, this also applies to *P. fijiensis*, as we did not find strains with reduced sensitivity and insertions in the promoter, but no mutations in the coding region. Isolates from wt populations lacked promoter insertions, but, occasionally, possessed mutations in the coding region.

We studied the regulatory nature of the inserted sequences in *P. fijiensis in silico* and showed that the 19‐bp (TAAATCTCGTACGATAGCA) repeat element is the most common feature. Within populations, we identified a clear genetic diversity in the number of promoter repeats. The frequency of isolates with more repeats was higher in banana plantations with up to eight DMI cycles, such as Cartagena, Zent and San Pablo. Although expected, it is also striking that all isolates from the untreated San Carlos plantation contained the single 19‐bp element. For the first time, using a targeted reverse genetics approach in *P. fijiensis*, we have validated that the presence of six copies of this element in the promoter increases the expression of *Pfcyp51* by at least three‐fold compared with wt isolates and other isolates with reduced sensitivity and up to three repeat elements. Previously, Cañas‐Gutiérrez *et al*. (2009) were unable to show such expression in experiments with *P. fijiensis* in response to propiconazole, and considered it to be either non‐existent or an unimportant mechanism in this fungus. However, this was probably because of the use of fewer isolates that showed a limited reduction in sensitivity. Hence, we now propose that promoter repeats constitute a genetic adaptation mechanism to the high selective pressure imposed on *P. fijiensis* by the continuous use of different DMI fungicides.

Although *P. fijiensis* is a difficult fungus to transform (Díaz‐Trujillo *et al*., 2011), and although site‐specific recombination levels seem to be very low, promoter swapping was successfully applied in our study. The introduction of the promoter from a *P. fijiensis* isolate with strongly reduced sensitivity into a sensitive isolate by site‐specific recombination resulted in a transformant with increased expression of *Pfcyp51*, and consequently reduced sensitivity to three azole fungicides, as a result of promoter replacement. The Swap 26 and Swap 121 transformants were at least four times less sensitive than the recipient wt isolate E22, but not as resistant as the wt resistant isolate Ca10_13 or the donor wt isolate Ca5_16, which had similar (Y136F and Y463D) coding domain mutations. Hence, we expect that the reverse experiment, replacing the wt promoter (with inserts) from an isolate with reduced sensitivity with a promoter from a sensitive wt, should result in an increase in sensitivity. Finally, swapping the wt *Pfcyp51* coding domain of a sensitive strain with this domain of an isolate with reduced sensitivity, thereby generating a strain with a wt coding domain, but multiple promoter inserts, which we have never encountered in nature, should result in increased sensitivity. However, the discovery of additional mechanisms for DMI sensitivity requires genetic studies, genome‐wide associations or mapping analyses (Chong‐Aguirre, 2016). We expect, however, that the combination of overexpression conferred by promoter insertions and *Pfcyp51* target site mutations will explain most DMI sensitivity modulations.

DMIs are and will probably remain a cornerstone of global black Sigatoka disease management. However, the risks of bad practices or excessive applications will exert a significant selection pressure on *P. fijiensis* populations, making these increasingly insensitive. Hence, DMI applications may lose their competitive advantage compared with other less environmentally friendly compounds. The practical spin‐off of this study is that we can now use a simple PCR assay to monitor, evaluate and predict reduced DMI sensitivity in *P. fijiensis* field populations. Although we have focused here on *P. fijiensis*, DMIs are evidently under pressure because of overall reduced sensitivity issues (Chen *et al*., 2016; Hayashi *et al*., 2002; Leroux and Walker, 2011; Liu *et al*., 2015; Mullins *et al*., 2011; Sun *et al*., 2013, 2014; Villani *et al*., 2016), and are therefore increasingly being studied in various other fungal pathogens (Alvarez‐Rueda *et al*., 2011; Becher and Wirsel, 2012; Carter *et al*., 2014; Cools *et al*., 2012; Frenkel *et al*., 2014; Li *et al*., 2012; Luo and Schnabel, 2008; Mair *et al*., 2016; Nikou *et al*., 2009; Rallos and Baudoin, 2016; Verweij *et al*., 2013). This fosters research and development for novel chemistry for efficient black Sigatoka control, although alternative products, such as the succinate dehydrogenase inhibitors (SDHIs) and QoIs, are also prone to resistance development (Arango Isaza *et al*., 2016; Scalliet *et al*., 2012). Therefore, disease management should, in the long run, embark on the availability of resistant banana germplasm. As this will take years, fungicide sensitivity monitoring and the strict adoption of application recommendations remain absolute necessities, irrespective of which banana cultivars dominate the export trade. A more science‐driven disease management and extension practice in global banana production is the prerequisite for a continuous production of this global fruit and major staple food.

## Experimental Procedures

### *Pseudocercospora fijiensis* isolates

A set of 25 monoascosporic *P. fijiensis* isolates from Africa, Asia and Latin America was used for fungicide sensitivity assays. Eight of the Latin American isolates were collected in Ecuador and 11 in Costa Rica (see Table 1). The larger set of Costa Rican isolates originated from four different banana plantations: Cartagena (Ca), Zent (Z), San Pablo (SP) and San Carlos (ZTSC) (see also Arango Isaza *et al*., 2016). The first three are frequently sprayed with fungicides, whereas the San Carlos plantation is in a plantain‐growing area with low *P. fijiensis* incidence, and hence fungicides are not required for disease control. We consider the *P. fijiensis* population from this area as a wt population. Isolates were obtained from CORBANA (Costa Rica), CIBE‐ESPOL (Ecuador) and the Westerdijk Fungal Biodiversity Institute (Africa and Asia).

### Determination of the *in vitro* sensitivity to DMI fungicides

The fungicides propiconazole, cyproconazole and difenoconazole were provided by Syngenta (Syngenta Crop Protection AG, Basel, Switzerland) and epoxiconazole was obtained from Sigma (Sigma‐Aldrich, St Louis, MO, USA). All compounds were of technical grade quality and were maintained in 100× stock solutions in either methanol or dimethylsulfoxide (DMSO). When applied to the culture medium, the final concentration of the solvents was <1% (v/v). For the initial *in vitro* sensitivity assays, the final concentrations tested for propiconazole were 10, 5.62, 3.16, 1.78, 1.0, 0.56 and 0.31 mg/L. Subsequently, to evaluate sensitive isolates more accurately, lower concentrations of fungicides were included in the assays (10.24, 2.56, 0.64, 0.16, 0.04, 0.016, 0.004 and 0 mg/L) and exploited to evaluate the performance of *P. fijiensis* transformants in the presence of propiconazole, difenoconazole and epoxiconazole.

The fungicide sensitivity of each isolate was determined by calculation of EC_50_. Quantitative analysis of fungal growth was determined by a modified 96‐well microtitre plate dilution assay (Montoya *et al*., 2006). Fifty microlitres of a 1 × 10^5^ mycelial parts/mL solution from each isolate were inoculated in 200 µL of potato dextrose broth (PDB) medium per well of a 96‐well polystyrene, flat‐bottomed, transparent plate (Corning, New York, USA; cat. # 3370). Plates were incubated at 25 °C in an incubator (Elbanton, Kerkdriel, the Netherlands) for 7 days before mycelial growth was measured. Each concentration was tested in duplicate per isolate, and per plate four blank controls were present. Individual plates were considered as one biological replicate, and tests were performed three times. Absorbance was initially measured at 620 nm in a TECAN A5082 plate reader (Männedorf, Switzerland) but, because of the variation of mycelial colours over the isolates, as well as the different colony morphologies, we eventually monitored growth at an absorbance of 690 nm in an Infinite® M200 PRO reader (TECAN), which enabled the measurement of higher sensitivities. The read design per well was settled at room temperature, leaving a border of 1000 µm, a bandwidth of 9 µm, circle‐filled reads of 21 read points (5 × 5, with no corner points for circle distribution) and each read point was measured five times. Read averages were plotted against days post‐inoculation (dpi) and compared with the other isolates and controls. The fungicide sensitivity of transformants and control isolates was determined in the aforementioned 96‐well polystyrene plates. Sealed plates were maintained at 27 °C in an incubator (Elbanton) in the dark and fungal growth was evaluated at 10 dpi. Plates were evaluated at 690 nm, whilst covered to reduce contamination. Data were analysed using GraphPad Prism7 (GraphPad Software, La Jolla, CA, USA).

### *Pfcyp51* coding domain and promoter amplification and sequencing

To amplify the *Pfcyp51* gene and the promoter region, specific primers located at the first repeat element and 22 bp upstream of the open reading frame (ORF) were used: *CYP51_Pfijien_*F1 (5′‐AAGGTCATATCGCAGG‐3′) and *CYP51_Pfijien_*R1 (5′‐GAATGTTATCGTGTGACA‐3′). A basic PCR mix was prepared and the PCR program consisted of 5 min of denaturation at 94 °C, followed by 34 cycles of 30 s at 94 °C, 30 s of annealing at 55 °C and 90 s of extension at 68 °C. An additional extension step of 7 min at 72 °C was performed at the end. DNA sequencing of the gene was performed at Macrogen (Seoul, Korea) and by the Genomics Facility of Wageningen University and Research (WUR), directly using the PCR products. To obtain the entire sequence of the gene and the promoter region, four primers were used in the sequencing reactions: *CYP51_Pfijien_*F2 (5′‐ACAGAAACATCACCTCC‐3′), CYP51_Pfijien_F3 (5′‐ATTGCTTCACTTTCATCC‐3′), *CYP51_Pfijien_*F4 (5′‐CTCTACCACGATCTCGAC‐3′) and *CYP51_Pfijien_*R2 (5′‐GATATGGATATAGTTGTC‐3′). The sequences obtained were assembled in contigs per isolate using CLC DNA Workbench software (CLC bio, Aarhus, Denmark) and the ORF was translated to amino acids and the protein sequences were aligned using the ClustalW plug in. The sequence alignments allowed the identification of mutations.

### *Pfcyp51* gene expression analysis

Extraction of total RNA was carried out with mycelia of *P. fijiensis* isolates grown for 10 days in PDB using the Qiagen RNA extraction plus mini kit (Qiagen Inc., Valencia, CA, USA). The integrity of the RNA was checked using agarose gel electrophoresis and the concentration was determined by measurement of the absorbance at 260 nm in a Nanodrop spectrophotometer (Thermo Scientific, Wilmington, MA, USA). Expression analysis was performed by qRT‐PCR using the primers QRTCYP‐forward: (5′‐CGCCAGTATTCGGCACAGATGTCG‐3′) and QRTCYP‐reverse: (5′‐TAACGTAGGACTGGAGGGCGGA‐3′), which amplify a fragment of 89 bp of the *Pfcyp51* gene, and primers QRTACT‐forward: (5′‐TCCGTCCTTGGTCTCGAATCTGGT‐3′) and QRTACT‐reverse: (5′‐TGCATACGGTCGGAGATACCTGGA‐3′), which amplify a fragment of 146 bp of the *P. fijiensis* actin gene which was used to normalize the expression. qRT‐PCRs were performed using 20 ng of total RNA per isolate in an Applied Biosystems ABI 7500 thermocycler (Waltham, MA, USA) using the Applied Biosystems Power SYBR® Green RNA‐to‐CT™ 1‐Step Kit, according to the manufacturer's instructions. The ΔΔCt method was used (with the actin gene as the endogenous control) to determine the level of *Pfcyp51* gene expression (Livak and Schmittgen, 2001).

### Analysis of promoter repeats of the *Pfcyp51* gene in four Costa Rican *P. fijiensis* populations

Genomic DNA (gDNA) samples of 225 *P. fijiensis* isolates from the four Costa Rican populations were analysed: 82 from the Cartagena population, 43 from the San Pablo population, 84 from the Zent population and 16 from the San Carlos wt population (Table S1, see Supporting Information). PCR fragments were amplified from gDNA using the specific primer pair, *P._fijiensis_repeats_*F (5′‐TCTCGTACGATAGCACCTGCCCA‐3′) and *P._fijiensis_repeats*_R (5′‐TGTTGGTGTAGGGGGTTAGGCCA‐3′), which was designed to amplify the promoter region of *Pfcyp51*. PCR conditions comprised 2 min at 95 °C, 30 cycles of 30 s of denaturation at 95 °C, 30 s of annealing at 68 °C and 2 min of extension at 72 °C, with an additional extension step of 10 min at 72 °C at the end of the reaction. PCR products were visualized and evaluated on 1% agarose gels and 11 isolates were selected for sequencing and subsequent analysis of promoter and coding sequences. Different repeat elements were aligned and a weblogo consensus sequence was generated (Crooks *et al*., 2004) to graph nucleotide conservation within the elements.

### Promoter swapping

We performed a promoter swapping experiment to test the effect of promoter repeats on *Pfcyp51* expression and, henceforward, on the sensitivity to several azole fungicides. The *Pfcyp51* donor promoter for homologous recombination was obtained from the resistant isolate Ca5_16. The recombination construct pPROM_CYP51_Ca5_16 comprised an upstream 2024‐bp fragment (the *PfCyp51* gene has an antisense position in the genome), obtained using primers 5‐CYP‐Prom Fwd (5′‐GGGGACAACTTTGTATAGAAAAGTTGAGGATATCAAGCACGCAC‐3′) and Rev (5′‐GGGGACTGCTTTTTTGTACAAACTTGGAAGAGAAACGGACTCCA‐3′), which was cloned in front of a cassette with the *hph* resistance gene and the GFP gene, followed by the upstream region of 1737 bp obtained with primers 3‐CYP‐Prom Fwd (5′‐GGGGACAGCTTTCTTGTACAAAGTGGGAATGAGCATTTGAGAGC‐3′) and Rev (5′‐GGGGACAACTTTGTATAATAAAGTTAATACTAGCGGAGGTTCG‐3′), containing the promoter region of isolate Ca5_16, which has six promoter repeats. Transformations were performed by *Agrobacterium tumefaciens*‐mediated transformation (Díaz‐Trujillo *et al*., 2011) using the sensitive wt *P. fijiensis* isolate E22, with a single repeat element and no mutations in the coding region. The promoter lengths of 250 GFP‐labelled transformants were compared with the promoter length of the resistant donor Ca5_16 and the sensitive recipient isolate E22. Transformants with a Ca5_16‐sized promoter were considered to be homologous recombinants; promoter swapped transformants were subsequently analysed for the integration of a 2629‐bp amplicon using PCR and employing the primers PROM‐HR‐30 Fwd (5′‐TGAGCATTTGAGAGC‐3′) and Rev (5′‐TTATGATCGCCTCCAAGC‐3′) located in the cassette and the *Pfcyp51* ORF, respectively.

## Supporting information

Additional Supporting Information may be found in the online version of this article at the publisher's website:

**Text S1** Genomic sequence of *Pfcyp51* in a set of 25 isolates of *Pseudocercospora fijiensis* from Asia, Africa and Latin America.

**Table S1** Analysis of *Pfcyp51* promoter repeats in 225 *Pseudocercospora fijiensis* isolates from Costa Rica, compared with 14 isolates from other countries.

**Fig. S1** Cross‐resistance between propiconazole and cyproconazole. The 50% inhibitory concentration (EC_50_) values were determined for both compounds on *Pseudocercospora fijiensis* colonies for the indicated strains at 10 days post‐inoculation (results are means of three independent experiments).
