# Supplementary material for: A new mechanism for reduced sensitivity to demethylation‐inhibitor fungicides in the fungal banana black Sigatoka pathogen Pseudocercospora fijiensis
Source: Mol Plant Pathol. 2018 Feb 13;19(6):1491–503. doi: 10.1111/mpp.12637 (PMC6637983; doi:10.1111/mpp.12637)
Supplement: Supplementary file 1 — Text S1 Genomic sequence of Pfcyp51 in a set of 25 isolates of Pseudocercospora fijiensis from Asia, Africa and Latin America. [file MPP-19-1491-s001.docx]

**Text S1** Genomic sequence of *Pfcyp51* in a set of 25 isolates of *Pseudocercospora fijiensis* from Asia, Africa and Latin America.

>C86

ATGCGGCGTGATGGTTGGACCCTTGGACCCTCGCAATTGATGAGAAGCAGGGGTGTCCCGTTCCTGCATGACTAGCGCC

AAAAAGTGACGCGACTGCCGAAATGTTAAATCTCGTACGATAGCACCTGCCCATGGACCACTCGAACATCACTGAAGGG

TAATCATTCAAGATCTTGGTGATTTGGCCTAACCCCCTACACCAACATCAACATCACTGACTCCGCGCAATGGGGCTCC

TCCAGGACGCCGCGGCGCTTTTCGACGCGCAATTTGGCCAGACAGCGACATGGAAACTAGTCCCCCTCGGCTTCAGCAT

CTTCTTCGCCGTATCCGTGTTGCTTAACGTGTTGCGCCAGCTGCTCTTCAGAAATCCAAACGAACCTCCGCTAGTATTC

CACTACGTGCCCTTCATTGGCAGCACTATCTCCTATGGCATCGACCCCTACAAGTTCTTCTTCGCCTGCCGTCAAAAAT

ACGGAGATTGCTTCACTTTCATCCTCCTTGGCAAGAAGACCACCGTGGTGCTGGGGACTAAAGGCAACGTCTTTATCTT

GAATGGAAAGCTCAAGGACGTCAATGCCGAGGAGATCTATAGCCCACTTACTACGCCAGTATTCGGCACAGATGTCGTC

TACGATTGTCCCAATTCGAAGCTCATGGAGCAGAAGAAGGTGCGTGCACACAATAAGCTGGATTTATGACAATGCTAAC

TTATACAGTTCGTCAAATATGGTCAGTGACTTGACTCCGCCGCGTCTGAGACAGCGCTCTCAACATGAGCTTCCACTTT

GGAGCGATACACTTGCTAACCTGCGCCTCCAGGCCTCACCTCCTCCGCCCTCCAGTCCTACGTTAAATTGATCACCAAA

GAGACCAAAGACTTCTTCTCCAAGGACAATCCAAGCAAGAAATTCGCATCCACCCACGGCACCGTCGACCTCCCGCCTG

CTATGGCTGAGCTTACTATCTACACCGCCAGCCGTTCGCTCCAGGGCAAAGAAGTCCGCGAAAAATTCGACTCCTCCTT

TGCCGACCTCTACCACGATCTCGACATGGGCTTCACTCCCATCAACTTCATGCTTCCATGGGCTCCACTGCCACAGAAT

AGAGCACGCGATCGCGCACAGAAGAAGATGGCGGAAGTCTACACAGCGATCATCAAAGAGCGACGCGAAAAGGGCGAGC

CTACTTCGGGAGAGAAAGAGCAGGACATGATTTGGAATCTGATGCAATGTCAGTACAAGAATGGTCAAGCAATTCCAGA

TAAGGAGATTGCGCACATGATGATTGCCCTTCTCATGGCTGGTCAACACTCGTCCTCGTCCACCTCATGCTGGATCCTT

CTCCGACTGGCTTCGCGACCAGATATCCAGGATGAGCTCCTTCAAGAACAGAAGGATGTGCTTGGCGTCAACGCAGATG

GATCAATCAAGGAGTTAACGTACGCCGACATCTCGCGCCTTCCACTCCTCAATCAAGTTGTCAAGGAGACACTCCGCCT

TCATGCTCCCATCCATTCTATTCTGCGACAAGTCAAGTCTCCGATGCCACTCGAAGGTACACCATACGTTGTCCCGACC

ACACACTCCCTCCTTGCTGCACCCGGTGCTACCTCACGAATGGACGAGCACTTCCCCGAAGCTATGCTGTGGGAACCCC

ACCGATGGGACGAGAACCCAAGTGAGAAGTACGCACATCTCGCACCAAAGCATGTCAAGGAGGGCGTCGCCGAAGAGAC

TGAAGATTACGGCTATGGTCTCGTCAGCAAAGGCGCCGCATCACCATATCTGCCATTCGGTGCTGGCCGACATAGATGC

ATCGGCGAGCAATTCGCCTATGTCCAGCTCCAGACCATCACCTCGGAAGTGATTCGCGATTTCAAGCTCTACAATGTCG

ACGGCAGCGACAAAGTTGTCGGCACAGATTACAGTTCGTTGTTCAGCAGACCTCTCTCGCCAGCCGTCGTGCGATGGGA

GAGGAGAGAAAAGAAATAGATTTCACAGTAAGAGTATGTTAATGCTAATCAGACAACTATATCCATATCTGCAGCTTCC

CTCCTCTGCGAGA

>Z4_7_Consensus

TGCAGATGATGGTTGGACCCTTGCGACCCTCGCAATTGA

TGAGAAGCAGGGGTGTCCCGTTCCTGCATGACTAGCGCCAAAAAGTGACGCGAATGCCGA

AATGTTAAATCTCGTACGATAGCACCTGCCCATGGACCAC

TCGAACCACACTGAAGGGTAATCATTCAAGATCTTGGTGATTTGGCCTAACCCCC

TACACCAACATCAACATCACTGACTCCGCGCAATGGGACTCCTCCAGGACGCCGCGGCGC

TTTTCGACGCGCAATTTGGCCAGATAGCGACATGGAAACTAGTCCCCCTCGGCTTCAGCA

TCTTCTTCGCCGTATCCGTGTTGCTTAACGTGTTGCGCCAGCTGCTTTTCAGAAATCCAA

ACGAACCTCCGCTAGTATTCCACTACGTGCCCTTCATTGGCAGCACTATCTCCTATGGCA

TCGACCCCTACAAGTTCTTCTTCGCCTGCCGTCAAAAATACGGAGATTGCTTCACTTTCA

TCCTCCTTGGCAAGAAGACCACCGTGGTGCTGGGGACTAAAGGCAACGACTTTATCTTGA

ATGGAAAGCTCAAGGACGTCAATGCCGAGGAGATCTATAGCCCACTTACTACGCCAGTAT

TCGGCACAGATGTCGTCTACGATTGTCCCAATTCGAAGCTCATGGAGCAGAAGAAGGTGC

GTGCACACAATAAGCTGGATTTATGACAATGCTAACTTATACAGTTCGTCAAATATGGTC

AGTGACTTGACTCCGCCGCGTCTGAGACAGCGCTCTCAACATGAGCTTCCACTTTGGAGC

GATACACTTGCTAACCTGCGCCTCCAGGCCTCACCTCCTCCGCCCTCCAGTCCTACGTTA

AATTGATCACCAAAGAGACCAAAGACTTCTTCTCCAAGGACAATCCAAGCAAGAAATTCG

CATCCACCCACGGCACCGTCGACCTCCCGCCTGCTATGGCTGAGCTTACTATCTACACCG

CCAGCCGTTCGCTCCAGGGCAAAGAAGTCCGCGAAAAATTCGACTCCTCCTTTGCCGACC

TCTACCACGATCTCGACATGGGCTTCACTCCCATCAACTTCATGCTTCCATGGGCTCCAC

TGCCACAGAATAGAGCACGCGATCGCGCACAGAAGAAGATGGCGGAAGTCTACACAGCG

ATCATCAAAGAGCGACGCGAAAAGGGCGAGCCTACTTCGGGAGAGAAAGAGCAGGACATG

ATTTGGAATCTGATGCAATGTCAGTACAAGAATGGTCAAGCAATTCCAGATAAGGAGATT

GCGCACATGATGATTGCCCTTCTCATGGGTGGTCAACACTCGTCCTCGTCCACCTCATGC

TGGATCCTTCTCCGACTGGCTTCGCGACCAGATATCCAGGATGAGCTCCTTCAAGAACAG

AAGGATGTGCTTGGCGTCAACGCAGATGGATCAATCAAGGAGTTAACGTACGCCGACATC

TCGCGCCTTCCACTCCTCAATCAAGTTGTCAAGGAGACACTCCGCCTTCATGCTCCCATC

CATTCTATTCTGCGACAAGTCAAGTCTCCGATGCCACTCGAAGGTACACCATACGTTGTC

CCGACCACACACTCCCTCCTTGCTGCACCCGGTGCTACCTCACGAATGGACGAGCACTTC

CCCGAAGCTATGCTGTGGGAACCCCACCGATGGGACGAGAACCCAAGTGAGAAGTACGCA

CATCTCGCACCAAAGCATGTCAAGGAGGGCGTCGCCGAAGAGACTGAAGATTACGGCTCT

GGTCTCGTCAGCAAAGGCGCCGCATCACCATATCTGCCATTCGGTGCTGGCCGACATAGA

TGCATCGGCGAGCAATTCGCCTATGTCCAGCTCCAGACCATCACCTCGGAAGTGATTCGC

GATTTCAAGCTCTACAATGTCGACGGCAGCGACAAAGTTGTCGGCACAGATTACAGTTCG

TTGTTCAGCAGACCTCTCTCGCCAGCCGTCGTGCGATGGGAGAGGAGAGAAAGAAATA

GATTTCACAGTA

>Z4_11_Consensus

GGTGCAGATGATGGTTGGACCCTTGGCACCCTCGCAATTGA

TGAGAAGCAGGGGTGTCCCGTTCCTGCATGACTAGCGCCAAAAAGTGACGCGAATGCCGA

AATGTTAAATCTCGTACGATAGCACCTGCCCATGGACCAC

TCGAACCACACTGAAGGGTAATCATTCAAGATCTTGGTGATTTGGCCTAACCCCC

TACACCAACATCAACATCACTGACTCCGCGCAATGGGACTCCTCCAGGACGCCGCGGCGC

TTTTCGACGCGCAATTTGGCCAGATAGCGACATGGAAACTAGTCCCCCTCGGCTTCAGCA

TCTTCTTCGCCGTATCCGTGTTGCTTAACGTGTTGCGCCAGCTGCTTTTCAGAAATCCAA

ACGAACCTCCGCTAGTATTCCACTACGTGCCCTTCATTGGCAGCACTATCTCCTATGGCA

TCGACCCCTACAAGTTCTTCTTCGCCTGCCGTCAAAAATACGGAGATTGCTTCACTTTCA

TCCTCCTTGGCAAGAAGACCACCGTGGTGCTGGGGACTAAAGGCAACGACTTTATCTTGA

ATGGAAAGCTCAAGGACGTCAATGCCGAGGAGATCTATAGCCCACTTACTACGCCAGTAT

TCGGCACAGATGTCGTCTACGATTGTCCCAATTCGAAGCTCATGGAGCAGAAGAAGGTGC

GTGCACACAATAAGCTGGATTTATGACAATGCTAACTTATACAGTTCGTCAAATATGGTC

AGTGACTTGACTCCGCCGCGTCTGAGACAGCGCTCTCAACATGAGCTTCCACTTTGGAGC

GATACACTTGCTAACCTGCGCCTCCNGGCCTCACCTCCTCCGCCCTCCAGTCCTACGTTA

AATTGATCACCAAAGAGACCAAAGACTTCTTCTCCAAGGACAATCCAAGCAAGAAATTCG

CATCCACCCACGGCACCGTCGACCTCCCGCCTGCTATGGCTGAGCTTACTATCTACACCG

CCAGCCGTTCGCTCCAGGGCAAAGAATTCCGCGAAAAATTCGACTCCTCCTTTGCCGACC

TCTACCACGATCTCGACATGGGCTTCACTCCCATCAACTTCATGCTTCCATGGGCTCCAC

TGCCACAGAAATAGAGCACGCGATCGCGCACAGAAGA-GATGGCGGAAGTCTACACAGCG

ATCATCAAAGAGCGACGCGAAAAGGGCGAGCCTACTTCGGGAGAGAAAGAGCAGGACATG

ATTTGGAATCTGATGCAATGTCAGTACAAGAATGGTCAAGCAATTCCAGATAAGGAGATT

GCGCACATGATGATTGCCCTTCTCATGGGTGGTCAACACTCGTCCTCGTCCACCTCATGC

TGGATCCTTCTCCGACTGGCTTCGCGACCAGATATCCAGGATGAGCTCCTTCAAGAACAG

AAGGATGTGCTTGGCGTCAACGCAGATGGATCAATCAAGGAGTTAACGTACGCCGACATC

TCGCGCCTTCCACTCCTCAATCAAGTTGTCAAGGAGACACTCCGCCTTCATGCTCCCATC

CATTCTATTCTGCGACAAGTCAAGTCTCCGATGCCACTCGAAGGTACACCATACGTTGTC

CCGACCACACACTCCCTCCTTGCTGCACCCGGTGCTACCTCACGAATGGACGAGCACTTC

CCCGAAGCTATGCTGTGGGAACCCCACCGATGGGACGAGAACCCAAGTGAGAAGTACGCA

CATCTCGCACCAAAGCATGTCAAGGAGGGCGTCGCCGAAGAGACTGAAGATTACGGCCAT

GGTCTCGTCAGCAAAGGCGCCGCATCACCATATCTGCCATTCGGTGCTGGCCGACATAGA

TGCATCGGCGAGCAATTCGCCTATGTCCAGCTCCAGACCATCACCTCGGAAGTGATTCGC

GATTTCAAGCTCTACAATGTCGACGGCAGCGACAAAGTTGTCGGCACAGATTACAGTTCG

TTGTTCAGCAGACCTCTCTCGCCAGCCGTCGTGCGATGGGAGAGGAGAGAAAAGAAATA

GATTTCACAGTAA

>Z4_14_Consensus

CGAGATGGTGCAGATGATGGTTGGACCCTTGGACCCTCGCAATTGA

TGAGAAGCAGGGGTGTCCCGTTCCTGCATGACTAGCGCCAAAAAGTGACGCGAATGCCGA

AATGTTAAATCTCGTACGATAGCACCTGCCCATGGACCAC

TCGAACCACACTGAAGGGTAATCATTCAAGATCTTGGTGATTTGGCCTAACCCCC

TACACCAACATCAACATCACTGACTCCGCGCAATGGGACTCCTCCAGGACGCCGCGGCGC

TTTTCGACGCGCAATTTGGCCAGATAGCGACATGGAAACTAGTCCCCCTCGGCTTCAGCA

TCTTCTTCGCCGTATCCGTGTTGCTTAACGTGTTGCGCCAGCTGCTTTTCAGAAATCCAA

ACGAACCTCCGCTAGTATTCCACTACGTGCCCTTCATTGGCAGCACTATCTCCTATGGCA

TCGACCCCTACAAGTTCTTCTTCGCCTGCCGTCAAAAATACGGAGATTGCTTCACTTTCA

TCCTCCTTGGCAAGAAGACCACCGTGGTGCTGGGGACTAAAGGCAACGACTTTATCTTGA

ATGGAAAGCTCAAGGACGTCAATGCCGAGGAGATCTATAGCCCACTTACTACGCCAGTAT

TCGGCACAGATGTCGTCTACGATTGTCCCAATTCGAAGCTCATGGAGCAGAAGAAGGTGC

GTGCACACAATAAGCTGGATTTATGACAATGCTAACTTATACAGTTCGTCAAATATGGTC

AGTGACTTGACTCCGCCGCGTCTGAGACAGCGCTCTCAACATGAGCTTCCACTTTGGAGC

GATACACTTGCTAACCTGCGCCTCCAGGCCTCACCTCCTCCGCCCTCCAGTCCTACGTTA

AATTGATCACCAAAGAGACCAAAGACTTCTTCTCCAAGGACAATCCAAGCAAGAAATTCG

CATCCACCCACGGCACCGTCGACCTCCCGCCTGCTATGGCTGAGCTTACTATCTACACCG

CCAGCCGTTCGCTCCAGGGCAAAGAAGTCCGCGAAAAATTCGACTCCTCCTTTGCCGACC

TCTACCACGATCTCGACATGGGCTTCACTCCCATCAACTTCATGCTTCCATGGGCTCCAC

TGCCACAGAATAGAGCACGCGATCGCGCACAGAAGAAGATGGCGGAAGTCTACACAGCG

ATCATCAAAGAGCGACGCGAAAAGGGCGAGCCTACTTCGGGAGAGAAAGAGCAGGACATG

ATTTGGAATCTGATGCAATGTCAGTACAAGAATGGTCAAGCAATTCCAGATAAGGAGATT

GCGCACATGATGATTGCCCTTCTCATGGGTGGTCAACACTCGTCCTCGTCCACCTCATGC

TGGATCCTTCTCCGACTGGCTTCGCGACCAGATATCCAGGATGAGCTCCTTCAAGAACAG

AAGGATGTGCTTGGCGTCAACGCAGATGGATCAATCAAGGAGTTAACGTACGCCGACATC

TCGCGCCTTCCACTCCTCAATCAAGTTGTCAAGGAGACACTCCGCCTTCATGCTCCCATC

CATTCTATTCTGCGACAAGTCAAGTCTCCGATGCCACTCGAAGGTACACCATACGTTGTC

CCGACCACACACTCCCTCCTTGCTGCACCCGGTGCTACCTCACGAATGGACGAGCACTTC

CCCGAAGCTATGCTGTGGGAACCCCACCGATGGGACGAGAACCCAAGTGAGAAGTACGCA

CATCTCGCACCAAAGCATGTCAAGGAGGGCGTCGCCGAAGAGACTGAAGATTACGGCGAT

GGTCTCGTCAGCAAAGGCGCCGCATCACCATATCTGCCATTCGGTGCTGGCCGACATAGA

TGCATCGGCGAGCAATTCGCCTATGTCCAGCTCCAGACCATCACCTCGGAAGTGATTCGC

GATTTCAAGCTCTACAATGTCGACGGCAGCGACAAAGTTGTCGGCACAGATTACAGTTCG

TTGTTCAGCAGACCTCTCTCGCCAGCCGTCGTGCGATGGGAGAGGAGAGAAAGAAATA

GATTTCACAGTA

>Z4_16_Consensus

CGAGATGGTGCAGATGATGGTTGGACCCTTGG-ACCCTCGCAATTGA

TGAGAAGCAGGGGTGTCCCGTTCCTGCATGACTAGCGCCAAAAAGTGACGCGAATGCCGA

AATGTTAAATCTCGTACGATAGCACCTGCCCATGGACCAC

TCGAACCACACTGAAGGGTAATCATTCAAGATCTTGGTGATTTGGCCTAACCCCC

TACACCAACATCAACATCACTGACTCCGCGCAATGGGACTCCTCCAGGACGCCGCGGCGC

TTTTCGACGCGCAATTTGGCCAGATAGCGACATGGAAACTAGTCCCCCTCGGCTTCAGCA

TCTTCTTCGCCGTATCCGTGTTGCTTAACGTGTTGCGCCAGCTGCTTTTCAGAAATCCAA

ACGAACCTCCGCTAGTATTCCACTACGTGCCCTTCATTGGCAGCACTATCTCCTATGGCA

TCGACCCCTACAAGTTCTTCTTCGCCTGCCGTCAAAAATACGGAGATTGCTTCACTTTCA

TCCTCCTTGGCAAGAAGACCACCGTGGTGCTGGGGACTAAAGGCAACGACTTTATCTTGA

ATGGAAAGCTCAAGGACGTCAATGCCGAGGAGATCTATAGCCCACTTACTACGCCAGTAT

TCGGCACAGATGTCGTCTACGATTGTCCCAATTCGAAGCTCATGGAGCAGAAGAAGGTGC

GTGCACACAATAAGCTGGATTTATGACAATGCTAACTTATACAGTTCGTCAAATATGGTC

AGTGACTTGACTCCGCCGCGTCTGAGACAGCGCTCTCAACATGAGCTTCCACTTTGGAGC

GATACACTTGCTAACCTGCGCCTCCAGGCCTCACCTCCTCCGCCCTCCAGTCCTACGTTA

AATTGATCACCAAAGAGACCAAAGACTTCTTCTCCAAGGACAATCCAAGCAAGAAATTCG

CATCCACCCACGGCACCGTCGACCTCCCGCCTGCTATGGCTGAGCTTACTATCTACACCG

CCAGCCGTTCGCTCCAGGGCAAAGAAGTCCGCGAAAAATTCGACTCCTCCTTTGCCGACC

TCTACCACGATCTCGACATGGGCTTCACTCCCATCAACTTCATGCTTCCATGGGCTCCAC

TGCCACAGAATAGAGCACGCGATCGCGCACAGAAGAAGATGGCGGAAGTCTACACAGCG

ATCATCAAAGAGCGACGCGAAAAGGGCGAGCCTACTTCGGGAGAGAAAGAGCAGGACATG

ATTTGGAATCTGATGCAATGTCAGTACAAGAATGGTCAAGCAATTCCAGATAAGGAGATT

GCGCACATGATGATTGCCCTTCTCATGGCTGGTCAACACTCGTCCTCGTCCACCTCATGC

TGGATCCTTCTCCGACTGGCTTCGCGACCAGATATCCAGGATGAGCTCCTTCAAGAACAG

AAGGATGTGCTTGGCGTCAACGCAGATGGATCAATCAAGGAGTTAACGTACGCCGACATC

TCGCGCCTTCCACTCCTCAATCAAGTTGTCAAGGAGACACTCCGCCTTCATGGTCCCATC

CATTCTATTCTGCGACAAGTCAAGTCTCCGATGCCACTCGAAGGTACACCATACGTTGTC

CCGACCACACACTCCCTCCTTGCTGCACCCGGTGCTACCTCACGAATGGACGAGCACTTC

CCCGAAGCTATGCTGTGGGAACCCCACCGATGGGACGAGAACCCAAGTGAGAAGTACGCA

CATCTCGCACCAAAGCATGTCAAGGAGGGCGTCGCCGAAGAGACTGAAGATTACGCCTAT

GGTCTCGTCAGCAAAGGCGCCGCATCACCATATCTGCCATTCGGTGCTGGCCGACATAGA

TGCATCGGCGAGCAATTCGCCTATGTCCAGCTCCAGACCATCACCTCGGAAGTGATTCGC

GATTTCAAGCTCTACAATGTCGACGGCAGCGACAAAGTTGTCGGCACAGATTACAGTTCG

TTGTTCAGCAGACCTCTCTCGCCAGCCGTCGTGCGATGGGAGAGGAGAGAAAAGAATA

GATTTCACAGTA

>Z8_12_Consensus

GCAGATGATGGTTGGACCCTTGG-ACCCTCGCAATTGA

TGAGAAGCAGGGGTGTCCCGTTCCTGCATGACTAGCGCCAAAAAGTGACGCGAATGCCGA

AATGTTAAATCTCGTACGATAGCACCTGCCCATCTC

GTACGATAGCACAAATGTTAAATCTCGTACGATAGCATGGACCAC

TCGAACCACACTGAAGGGTAATCATTCAAGATCTTGGTGATTTGGCCTAACCCCC

TACACCAACATCAACATCACTGACTCCGCGCAATGGGACTCCTCCAGGACGCCGCGGCGC

TTTTCGACGCGCAATTTGGCCAGATAGCGACATGGAAACTAGTCCCCCTCGGCTTCAGCA

TCTTCTTCGCCGTATCCGTGTTGCTTAACGTGTTGCGCCAGCTGCTTTTCAGAAATCCAA

ACGAACCTCCGCTAGTATTCCACTACGTGCCCTTCATTGGCAGCACTATCTCCTATGGCA

TCGACCCCTACAAGTTCTTCTTCGCCTGCCGTCAAAAATACGGAGATTGCTTCACTTTCA

TCCTCCTTGGCAAGAAGACCACCGTGGTGCTGGGGACTAAAGGCAACGACTTTATCTTGA

ATGGAAAGCTCAAGGACGTCAATGCCGAGGAGATCTATAGCCCACTTACTACGCCAGTAT

TCGGCACAGATGTCGTCTTTGATTGTCCCAATTCGAAGCTCATGGAGCAGAAGAAGGTGC

GTGCACACAATAAGCTGGATTTATGACAATGCTAACTTATACAGTTCGTCAAATATGGTC

AGTGACTTGACTCCGCCGCGTCTGAGACAGCGCTCTCAACATGAGCTTCCACTTTGGAGC

GATACACTTGCTAACCTGCGCCTCCAGGCCTCACCTCCTCCGCCCTCCAGTCCTACGTTA

AATTGATCACCAAAGAGACCAAAGACTTCTTCTCCAAGGACAATCCAAGCAAGAAATTCG

CATCCACCCACGGCACCGTCGACCTCCCGCCTGCTATGGCTGAGCTTACTATCTACACCG

CCAGCCGTTCGCTCCAGGGCAAAGAAGTCCGCGAAAAATTCGACTCCTCCTTTGCCGACC

TCTACCACGATCTCGACATGGGCTTCACTCCCATCAACTTCATGCTTCCATGGGCTCCAC

TGCCACAGAATAGAGCACGCGATCGCGCACAGAAGAAGATGGCGGAAGTCTACACAGCG

ATCATCAAAGAGCGACGCGAAAAGGGCGAGCCTACTTCGGGAGAGAAAGAGCAGGACATG

ATTTGGAATCTGATGCAATGTCAGTACAAGAATGGTCAAGCAATTCCAGATAAGGAGATT

GCGCACATGATGATTGCCCTTCTCATGGCTGGTCAACACTCGTCCTCGTCCACCTCATGC

TGGATCCTTCTCCGACTGGCTTCGCGACCAGATATCCAGGATGAGCTCCTTCAAGAACAG

AAGGATGTGCTTGGCGTCAACGCAGATGGATCAATCAAGGAGTTAACGTACGCCGACATC

TCGCGCCTTCCACTCCTCAATCAAGTTGTCAAGGAGACACTCCGCCTTCATGCTCCCATC

CATTCTATTCTGCGACAAGTCAAGTCTCCGATGCCACTCGAAGGTACACCATACGTTGTC

CCGACCACACACTCCCTCCTTGCTGCACCCGGTGCTACCTCACGAATGGACGAGCACTTC

CCCGAAGCTATGCTGTGGGAACCCCACCGATGGGACGAGAACCCAAGTGAGAAGTACGCA

CATCTCGCACCAAAGCATGTCAAGGAGGGCGTCGCCGAAGAGACTGAAGATTACGGCGAT

GGTCTCGTCAGCAAAGGCGCCGCATCACCATATCTGCCATTCGGTGCTGGCCGACATAGA

TGCATCGGCGAGCAATTCGCCTATGTCCAGCTCCAGACCATCACCTCGGAAGTGATTCGC

GATTTCAAGCTCTACAATGTCGACGGCAGCGACAAAGTTGTCGGCACAGATTACAGTTCG

TTGTTCAGCAGACCTCTCTCGCCAGCCGTCGTGCGATGGGAGAGGAGAGAAAAGAAATA

GATTTCACAGTAAGA

>Z8_17_Consensus

ATGATGGTTGGACCCTTGGACCCTCGCAATTGA

TGAGAAGCAGGGGTGTCCCGTTCCTGCATGACTAGCGCCAAAAAGTGACGCGAATGCCGA

AATGTTAAATCTCGTACGATAGCACCTGCCCATGGACCAC

TCGAACCACACTGAAGGGTAATCATTCAAGATCTTGGTGATTTGGCCTAACCCCC

TACACCAACATCAACATCACTGACTCCGCGCAATGGGACTCCTCCAGGACGCCGCGGCGC

TTTTCGACGCGCAATTTGGCCAGATAGCGACATGGAAACTAGTCCCCCTCGGCTTCAGCA

TCTTCTTCGCCGTATCCGTGTTGCTTAACGTGTTGCGCCAGCTGCTTTTCAGAAATCCAA

ACGAACCTCCGCTAGTATTCCACTACGTGCCCTTCATTGGCAGCACTATCTCCTATGGCA

TCGACCCCTACAAGTTCTTCTTCGCCTGCCGTCAAAAATACGGAGATTGCTTCACTTTCA

TCCTCCTTGGCAAGAAGACCACCGTGGTGCTGGGGACTAAAGGCAACGACTTTATCTTGA

ATGGAAAGCTCAAGGACGTCAATGCCGAGGAGATCTATAGCCCACTTACTACGCCAGTAT

TCGGCACAGATGTCGTCTACGATTGTCCCAATTCGAAGCTCATGGAGCAGAAGAAGGTGC

GTGCACACAATAAGCTGGATTTATGACAATGCTAACTTATACAGTTCGTCAAATATGGTC

AGTGACTTGACTCCGCCGCGTCTGAGACAGCGCTCTCAACATGAGCTTCCACTTTGGAGC

GATACACTTGCTAACCTGCGCCTCCAGGCCTCACCTCCTCCGCCCTCCAGTCCTACGTTA

AATTGATCACCAAAGAGACCAAAGACTTCTTCTCCAAGGACAATCCAAGCAAGAAATTCG

CATCCACCCACGGCACCGTCGACCTCCCGCCTGCTATGGCTGAGCTTACTATCTACACCG

CCAGCCGTTCGCTCCAGGGCAAAGAAGTCCGCGAAAAATTCGACTCCTCCTTTGCCGACC

TCTACCACGATCTCGACATGGGCTTCACTCCCATCAACTTCATGCTTCCATGGGCTCCAC

TGCCACAGAATAGAGCACGCGATCGCGCACAGAAGAAGATGGCGGAAGTCTACACAGCG

ATCATCAAAGAGCGACGCGAAAAGGGCGAGCCTACTTCGGGAGAGAAAGAGCAGGACATG

ATTTGGAATCTGATGCAATGTCAGTACAAGAATGGTCAAGCAATTCCAGATAAGGAGATT

GCGCACATGATGATTGCCCTTCTCATGGGTGGTCAACACTCGTCCTCGTCCACCTCATGC

TGGATCCTTCTCCGACTGGCTTCGCGACCAGATATCCAGGATGAGCTCCTTCAAGAACAG

AAGGATGTGCTTGGCGTCAACGCAGATGGATCAATCAAGGAGTTAACGTACGCCGACATC

TCGCGCCTTCCACTCCTCAATCAAGTTGTCAAGGAGACACTCCGCCTTCATGCTCCCATC

CATTCTATTCTGCGACAAGTCAAGTCTCCGATGCCACTCGAAGGTACACCATACGTTGTC

CCGACCACACACTCCCTCCTTGCTGCACCCGGTGCTACCTCACGAATGGACGAGCACTTC

CCCGAAGCTATGCTGTGGGAACCCCACCGATGGGACGAGAACCCAAGTGAGAAGTACGCA

CATCTCGCACCAAAGCATGTCAAGGAGGGCGTCGCCGAAGAGACTGAAGATTACGGCTCT

GGTCTCGTCAGCAAAGGCGCCGCATCACCATATCTGCCATTCGGTGCTGGCCGACATAGA

TGCATCGGCGAGCAATTCGCCTATGTCCAGCTCCAGACCATCACCTCGGAAGTGATTCGC

GATTTCAAGCTCTACAATGTCGACGGCAGCGACAAAGTTGTCGGCACAGATTACAGTTCG

TTGTTCAGCAGACCTCTCTCGCCAGCCGTCGTGCGATGGGAGAGGAGAGAAAAGAAATA

GATTTCACAGTAAGAG

>Z8_18_Consensus

TTGG-ACCCTCGCAATTGA

TGAGAAGCAGGGGTGTCCCGTTCCTGCATGACTAGCGCCAAAAAGTGACGCGAATGCCGA

AATGTTAAATCTCGTACGATAGCACCTGCCCATCTC

GTACGATAGCACAAATGTTAAATCTCGTACGATAGCATGGACCAC

TCGAACCACACTGAAGGGTAATCATTCAAGATCTTGGTGATTTGGCCTAACCCCC

TACACCAACATCAACATCACTGACTCCGCGCAATGGGACTCCTCCAGGACGCCGCGGCGC

TTTTCGACGCGCAATTTGGCCAGATAGCGACATGGAAACTAGTCCCCCTCGGCTTCAGCA

TCTTCTTCGCCGTATCCGTGTTGCTTAACGTGTTGCGCCAGCTGCTTTTCAGAAATCCAA

ACGAACCTCCGCTAGTATTCCACTACGTGCCCTTCATTGGCAGCACTATCTCCTATGGCA

TCGACCCCTACAAGTTCTTCTTCGCCTGCCGTCAAAAATACGGAGATTGCTTCACTTTCA

TCCTCCTTGGCAAGAAGACCACCGTGGTGCTGGGGACTAAAGGCAACGACTTTATCTTGA

ATGGAAAGCTCAAGGACGTCAATGCCGAGGAGATCTATAGCCCACTTACTACGCCAGTAT

TCGGCACAGATGTCGTCTTTGATTGTCCCAATTCGAAGCTCATGGAGCAGAAGAAGGTGC

GTGCACACAATAAGCTGGATTTATGACAATGCTAACTTATACAGTTCGTCAAATATGGTC

AGTGACTTGACTCCGCCGCGTCTGAGACAGCGCTCTCAACATGAGCTTCCACTTTGGAGC

GATACACTTGCTAACCTGCGCCTCCAGGCCTCACCTCCTCCGCCCTCCAGTCCTACGTTA

AATTGATCACCAAAGAGACCAAAGACTTCTTCTCCAAGGACAATCCAAGCAAGAAATTCG

CATCCACCCACGGCACCGTCGACCTCCCGCCTGCTATGGCTGAGCTTACTATCTACACCG

CCAGCCGTTCGCTCCAGGGCAAAGAAGTCCGCGAAAAATTCGACTCCTCCTTTGCCGACC

TCTACCACGATCTCGACATGGGCTTCACTCCCATCAACTTCATGCTTCCATGGGCTCCAC

TGCCACAGAATAGAGCACGCGATCGCGCACAGAAGAAGATGGCGGAAGTCTACACAGCG

ATCATCAAAGAGCGACGCGAAAAGGGCGAGCCTACTTCGGGAGAGAAAGAGCAGGACATG

ATTTGGAATCTGATGCAATGTCAGTACAAGAATGGTCAAGCAATTCCAGATAAGGAGATT

GCGCACATGATGATTGCCCTTCTCATGGCTGGTCAACACTCGTCCTCGTCCACCTCATGC

TGGATCCTTCTCCGACTGGCTTCGCGACCAGATATCCAGGATGAGCTCCTTCAAGAACAG

AAGGATGTGCTTGGCGTCAACGCAGATGGATCAATCAAGGAGTTAACGTACGCCGACATC

TCGCGCCTTCCACTCCTCAATCAAGTTGTCAAGGAGACACTCCGCCTTCATGCTCCCATC

CATTCTATTCTGCGACAAGTCAAGTCTCCGATGCCACTCGAAGGTACACCATACGTTGTC

CCGACCACACACTCCCTCCTTGCTGCACCCGGTGCTACCTCACGAATGGACGAGCACTTC

CCCGAAGCTATGCTGTGGGAACCCCACCGATGGGACGAGAACCCAAGTGAGAAGTACGCA

CATCTCGCACCAAAGCATGTCAAGGAGGGCGTCGCCGAAGAGACTGAAGATTACGGCGAT

GGTCTCGTCAGCAAAGGCGCCGCATCACCATATCTGCCATTCGGTGCTGGCCGACATAGA

TGCATCGGCGAGCAATTCGCCTATGTCCAGCTCCAGACCATCACCTCGGAAGTGATTCGC

GATTTCAAGCTCTACAATGTCGACGGCAGCGACAAAGTTGTCGGCACAGATTACAGTTCG

TTGTTCAGCAGACCTCTCTCGCCAGCCGTCGTGCGATGGGAGAGGAGAGAAAGAAATA

GATTTCACAGTAAGAG

>Ca1_5_Cyp51

CCAAAAAAGTGACGCGAATGCCGAAATGTTAAAATCTCGTACGATAGCACCTCCCCATCTCGTACGATAG

CACAAATGTTAAATCTCGTACGATAGCATGGACCACTCGAACCCACACTGAAGGGTAATCATTCAAGATC

TTGGTGATTTGGCCTAACCCCCTACACCAACATCAACATCACTGACTCCGCGCAATGGGACTCCTCCAGG

ACGCCGCGGCGCTTTTCGACGCGCAATTTGGCCAGATAGCGACATGGAAACTAGTCCCCCTCGGCTTCAG

CATCTTCTTCGCCGTATCCGTGTTGCTTAACGTGTTGCGCCAGCTGCTTTTCAGAAATCCAAACGAACCT

CCGCTAGTATTCCACTACGTGCCCTTCATTGGCAGCACTATCTCCTATGGCATCGACCCCTACAAGTTCT

TCTTCGCCTGCCGTCAAAAATACGGAGATTGCTTCACTTTCATCCTCCTTGGCAAGAAGACCACCGTGGT

GCTGGGGACTAAAGGCAACGACTTTATCTTGAATGGAAAGCTCAAGGACGTCAATGCCGAGGAGATCTAT

AGCCCACTTACTACGCCAGTATTCGGCACAGATGTCGTCTTTGATTGTCCCAATTCGAAGCTCATGGAGC

AGAAGAAGGTGCGTGCACACAATAAGCTGGATTTATGACAATGCTAACTTATACAGTTCGTCAAATATGG

TCAGTGACTTGACTCCGCCGCGTCTGAGACAGCGCTCTCAACATGAGCTTCCACTTTGGAGCGATACACT

TGCTAACCTGCGCCTCCAGGCCTCACCTCCTCCGCCCTCCAGTCCTACGTTAAATTGATCACCAAAGAGA

CCAAAGACTTCTTCTCCAAGGACAATCCAAGCAAGAAATTCGCATCCACCCACGGCACCGTCGACCTCCC

GCCTGCTATGGCTGAGCTTACTATCTACACCGCCAGCCGTTCGCTCCAGGGCAAAGAAGTCCGCGAAAAA

TTCGACTCCTCCTTTGCCGACCTCTACCACGATCTCGACATGGGCTTCACTCCCATCAACTTCATGCTTC

CATGGGCTCCACTGCCACAGAATAGAGCACGCGATCGCGCACAGAAGAAGATGGCGGAAGTCTACACAGC

GATCATCAAAGAGCGACGCGAAAAGGGCGAGCCTACTTCGGGAGAGAAAGAGCAGGACATGATTTGGAAT

CTGATGCAATGTCAGTACAAGAATGGTCAAGCAATTCCAGATAAGGAGATTGCGCACATGATGATTGCCC

TTCTCATGGCTGGTCAACACTCGTCCTCGTCCACCTCATGCTGGATCCTTCTCCGACTGGCTTCGCGACC

AGATATCCAGGATGAGCTCCTTCAAGAACAGAAGGATGTGCTTGGCGTCAACGCAGATGGATCAATCAAG

GAGTTAACGTACGCCGACATCTCGCGCCTTCCACTCCTCAATCAAGTTGTCAAGGAGACACTCCGCCTTC

ATGGTCCCATCCATTCTATTCTGCGACAAGTCAAGTCTCCGATGCCACTCGAAGGTACACCATACGTTGT

CCCGACCACACACTCCCTCCTTGCTGCACCCGGTGCTACCTCACGAATGGACGAGCACTTCCCCGAAGCT

ATGCTGTGGGAACCCCACCGATGGGACGAGAACCCAAGTGAGAAGTACGCACATCTCGCACCAAAGCATG

TCAAGGAGGGCGTCGCCGAAGAGACTGAAGATTACGGCGATGGTCTCGTCAGCAAAGGCGCCGCATCACC

ATATCTGCCATTCGGTGCTGGCCGACATAGATGCATCGGCGAGCAATTCGCCTATGTCCAGCTCCAGACC

ATCACCTCGGAAGTGATTCGCGATTTCAAGCTCTACAATGTCGACGGCAGCGACAAAGTTGTCGGCACAG

ATTACAGTTCGTTGTTCAGCAGACCTCTCTCGCCAGCCGTCGTGCGATGGGAGAGGAGAGAAAAGAAATA

GATTTCACAGTAAGAGTATGTTAATGCTAATCAGACAACTATATCCATATCTGCAGCTTCCCTCCATCTG

TCACACGATAACATTCTATAAGAAGAACCAACCTGCAGCGCACTACCAACG

>Ca5_16_Consensus

GAGATGGTGCAGATGATGGTTGGACCCTTGG-ACCCTCGCAATTGA

TGAGAAGCAGGGGTGTCCCGTTCCTGCATGACTAGCGCCAAAAAGTGACGCGAATGCCGA

AATGTTAAATCTCGTACGATAGCACCTGCCCATAAATCTCGTACGATAGCATAAAATCTC

GTACGATAGCATAAAATCTCGTACGATGTTAAATCTCGTACGATAGCATAAATCTCGTAC

GATAGCACCTGCCCATTGAAGGGTAATCATTCAAGATCTTGGTGATTTGGCCTAACCCCC

TACACCAACATCAACATCACTGACTCCGCGCAATGGGACTCCTCCAGGACGCCGCGGCGC

TTTTCGACGCGCAATTTGGCCAGATAGCGACATGGAAACTAGTCCCCCTCGGCTTCAGCA

TCTTCTTCGCCGTATCCGTGTTGCTTAACGTGTTGCGCCAGCTGCTTTTCAGAAATCCAA

ACGAACCTCCGCTAGTATTCCACTACGTGCCCTTCATTGGCAGCACTATCTCCTATGGCA

TCGACCCCTACAAGTTCTTCTTCGCCTGCCGTCAAAAATACGGAGATTGCTTCACTTTCA

TCCTCCTTGGCAAGAAGACCACCGTGGTGCTGGGGACTAAAGGCAACGACTTTATCTTGA

ATGGAAAGCTCAAGGACGTCAATGCCGAGGAGATCTATAGCCCACTTACTACGCCAGTAT

TCGGCACAGATGTCGTCTTCGATTGTCCCAATTCGAAGCTCATGGAGCAGAAGAAGGTGC

GTGCACACAATAAGCTGGATTTATGACAATGCTAACTTATACAGTTCGTCAAATATGGTC

AGTGACTTGACTCCGCCGCGTCTGAGACAGCGCTCTCAACATGAGCTTCCACTTTGGAGC

GATACACTTGCTAACCTGCGCCTCCAGGCCTCACCTCCTCCGCCCTCCAGTCCTACGTTA

AATTGATCACCAAAGAGACCAAAGACTTCTTCTCCAAGGACAATCCAAGCAAGAAATTCG

CATCCACCCACGGCACCGTCGACCTCCCGCCTGCTATGGCTGAGCTTACTATCTACACCG

CCAGCCGTTCGCTCCAGGGCAAAGAAGTCCGCGAAAAATTCGACTCCTCCTTTGCCGACC

TCTACCACGATCTCGACATGGGCTTCACTCCCATCAACTTCATGCTTCCATGGGCTCCAC

TGCCACAGAA-TAGAGCACGCGATCGCGCACAGAAGAAGATGGCGGAAGTCTACACAGCG

ATCATCAAAGAGCGACGCGAAAAGGGCGAGCCTACTTCGGGAGAGAAAGAGCAGGACATG

ATTTGGAATCTGATGCAATGTCAGTACAAGAATGGTCAAGCAATTCCAGATAAGGAGATT

GCGCACATGATGATTGCCCTTCTCATGGCTGGTCAACACTCGTCCTCGTCCACCTCATGC

TGGATCCTTCTCCGACTGGCTTCGCGACCAGATATCCAGGATGAGCTCCTTCAAGAACAG

AAGGATGTGCTTGGCGTCAACGCAGATGGATCAATCAAGGAGTTAACGTACGCCGACATC

TCGCGCCTTCCACTCCTCAATCAAGTTGTCAAGGAGACACTCCGCCTTCATGCTCCCATC

CATTCTATTCTGCGACAAGTCAAGTCTCCGATGCCACTCGAAGGTACACCATACGTTGTC

CCGACCACACACTCCCTCCTTGCTGCACCCGGTGCTACCTCACGAATGGACGAGCACTTC

CCCGAAGCTATGCTGTGGGAACCCCACCGATGGGACGAGAACCCAAGTGAGAAGTACGCA

CATCTCGCACCAAAGCATGTCAAGGAGGGCGTCGCCGAAGAGACTGAAGATTACGGCGAT

GGTCTCGTCAGCAAAGGCGCCGCATCACCATATCTGCCATTCGGTGCTGGCCGACATAGA

TGCATCGGCGAGCAATTCGCCTATGTCCAGCTCCAGACCATCACCTCGGAAGTGATTCGC

GATTTCAAGCTCTACAATGTCGACGGCAGCGACAAAGTTGTCGGCACAGATTACAGTTCG

TTGTTCAGCAGACCTCTCTCGCCAGCCGTCGTGCGAT-GGGAGAGGAGAGAAAGAAA-TA

GATTTCACAGTAAGAGTAT

>Ca6_11-Consensus

GCAGATGATGGTTGGACCCTTGG-ACCCTCGCAATTGA

TGAGAAGCAGGGGTGTCCCGTTCCTGCATGACTAGCGCCAAAAAGTGACGCGAATGCCGA

AATGTTAAATCTCGTACGATAGCACCTGCCCATAAATCTCGTACGATAGCATAAAATCTC

GTACGATAGCATAAAATCTCGTACGATGTTAAATCTCGTACGATAGCATAAATCTCGTAC

GATAGCACCTGCCCATTGAAGGGTAATCATTCAAGATCTTGGTGATTTGGCCTAACCCCC

TACACCAACATCAACATCACTGACTCCGCGCAATGGGACTCCTCCAGGACGCCGCGGCGC

TTTTCGACGCGCAATTTGGCCAGATAGCGACATGGAAACTAGTCCCCCTCGGCTTCAGCA

TCTTCTTCGCCGTATCCGTGTTGCTTAACGTGTTGCGCCAGCTGCTTTTCAGAAATCCAA

ACGAACCTCCGCTAGTATTCCACTACGTGCCCTTCATTGGCAGCACTATCTCCTATGGCA

TCGACCCCTACAAGTTCTTCTTCGCCTGCCGTCAAAAATACGGAGATTGCTTCACTTTCA

TCCTCCTTGGCAAGAAGACCACCGTGGTGCTGGGGACTAAAGGCAACGACTTTATCTTGA

ATGGAAAGCTCAAGGACGTCAATGCCGAGGAGATCTATAGCCCACTTACTACGCCAGTAT

TCGGCACAGATGTCGTCTTCGATTGTCCCAATTCGAAGCTCATGGAGCAGAAGAAGGTGC

GTGCACACAATAAGCTGGATTTATGACAATGCTAACTTATACAGTTCGTCAAATATGGTC

AGTGACTTGACTCCGCCGCGTCTGAGACAGCGCTCTCAACATGAGCTTCCACTTTGGAGC

GATACACTTGCTAACCTGCGCCTCCAGGCCTCACCTCCTCCGCCCTCCAGTCCTACGTTA

AATTGATCACCAAAGAGACCAAAGACTTCTTCTCCAAGGACAATCCAAGCAAGAAATTCG

CATCCACCCACGGCACCGTCGACCTCCCGCCTGCTATGGCTGAGCTTACTATCTACACCG

CCAGCCGTTCGCTCCAGGGCAAAGAAGTCCGCGAAAAATTCGACTCCTCCTTTGCCGACC

TCTACCACGATCTCGACATGGGCTTCACTCCCATCAACTTCATGCTTCCATGGGCTCCAC

TGCCACAGAA-TAGAGCACGCGATCGCGCACAGAAGAAGATGGCGGAAGTCTACACAGCG

ATCATCAAAGAGCGACGCGAAAAGGGCGAGCCTACTTCGGGAGAGAAAGAGCAGGACATG

ATTTGGAATCTGATGCAATGTCAGTACAAGAATGGTCAAGCAATTCCAGATAAGGAGATT

GCGCACATGATGATTGCCCTTCTCATGGCTGGTCAACACTCGTCCTCGTCCACCTCATGC

TGGATCCTTCTCCGACTGGCTTCGCGACCAGATATCCAGGATGAGCTCCTTCAAGAACAG

AAGGATGTGCTTGGCGTCAACGCAGATGGATCAATCAAGGAGTTAACGTACGCCGACATC

TCGCGCCTTCCACTCCTCAATCAAGTTGTCAAGGAGACACTCCGCCTTCATGCTCCCATC

CATTCTATTCTGCGACAAGTCAAGTCTCCGATGCCACTCGAAGGTACACCATACGTTGTC

CCGACCACACACTCCCTCCTTGCTGCACCCGGTGCTACCTCACGAATGGACGAGCACTTC

CCCGAAGCTATGCTGTGGGAACCCCACCGATGGGACGAGAACCCAAGTGAGAAGTACGCA

CATCTCGCACCAAAGCATGTCAAGGAGGGCGTCGCCGAAGAGACTGAAGATTACGGCGAT

GGTCTCGTCAGCAAAGGCGCCGCATCACCATATCTGCCATTCGGTGCTGGCCGACATAGA

TGCATCGGCGAGCAATTCGCCTATGTCCAGCTCCAGACCATCACCTCGGAAGTGATTCGC

GATTTCAAGCTCTACAATGTCGACGGCAGCGACAAAGTTGTCGGCACAGATTACAGTTCG

TTGTTCAGCAGACCTCTCTCGCCAGCCGTCGTGCGAT-GGGAGAGGAGAGAAAAGAAATA

GA

>Ca10_13

GATGGTGCAGATGATGGTTGGACCCTTGGACCCTCGCAATTGATGAGAAGCAGGGGTGTCCCGTTCCTGCATGACTAGC

GCCAAAAAGTGACGCGAATGCCGAAATGTTAAATCTCGTACGATAGCACCTGCCCATAAATCTCGTACGATAGCATAAA

ATCTCGTACGATAGCATAAAATCTCGTACGATGTTAAATCTCGTACGATAGCATAAATCTCGTACGATAGCACCTGCCC

ATTGAAGGGTAATCATTCAAGATCTTGGTGATTTGGCCTAACCCCCTACACCAACATCAACATCACTGACTCCGCGCAA

TGGGACTCCTCCAGGACGCCGCGGCGCTTTTCGACGCGCAATTTGGCCAGATAGCGACATGGAAACTAGTCCCCCTCGG

CTTCAGCATCTTCTTCGCCGTATCCGTGTTGCTTAACGTGTTGCGCCAGCTGCTTTTCAGAAATCCAAACGAACCTCCG

CTAGTATTCCACTACGTGCCCTTCATTGGCAGCACTATCTCCTATGGCATCGACCCCTACAAGTTCTTCTTCGCCTGCC

GTCAAAAATACGGAGATTGCTTCACTTTCATCCTCCTTGGCAAGAAGACCACCGTGGTGCTGGGGACTAAAGGCAACGA

CTTTATCTTGAATGGAAAGCTCAAGGACGTCAATGCCGAGGAGATCTATAGCCSACTTACTACGCCAGTATTCGGCACA

GATGTCRTCTTCGATTGTCCCAATTCGAAGCTCATGGAGCGGAGGAAGGTGCGTGCACACAATAAGCTGGATTTATGAC

AATGCTAACTTATACAGTTCGTCAAATATGGTCAGTGACTTGACTCCGCCGCGTCTGAGACAGCGCTCTCAACATGAGC

TTCCACTTTGGAGCGATACACTTGCTAACCTGCGCCTCCAGGCCTCACCTCCTCCGCCCTCCAGTCCTACGTTAAATTG

ATCACCAAAGAGACCAAAGACTTCTTCTCCAAGGACAATCCAAGCAAGAAATTCGCATCCACCCACGGCACCGTCGACC

TCCCGCCTGCTATGGCTGAGCTTACTATCTACACCGCCAGCCGTTCGCTCCAGGGCAAAGAAGTCCGCGAAAAATTCGA

CTCCTCCTTTGCCGACCTCTACCACGATCTCGACATGGGCTTCACTCCCATCAACTTCATGCTTCCATGGGCTCCACTG

CCACAGAATAGAGCACGCGATCGCGCACAGAAGAAGATGGCGGAAGTCTACACAGCGATCATCAAAGAGCGACGCGAAA

AGGGCGAGCCTACTTCGGGAGAGAAAGAGCAGGACATGATTTGGAATCTGATGCAaTGTCAGTACAAGAATGGTCAAGC

AATTCCAGATAAGGAGATTGCGCACATGATGATTGCCCTTCTCATGGCTGGTCAACACTCGTCCTCGTCCACCTCATGC

TGGATCCTTCTCCGACTGGCTTCGCGACCAGATATCCAGGATGAGCTCCTTCAAGAACAGAAGGATGTGCTTGGCGTCA

ACGCAGATGGATCAATCAAGGAGTTAACGTACGCCGACATCTCGCGCCTTCCACTCCTCAATCAAGTTGTCAAGGAGAC

ACTCCGCCTTCATGCTCCCATCCATTCTATTCTGCGACAAGTCAAGTCTCCGATGCCACTCGAAGGTACACCATACGTT

GTCCCGACCACACACTCCCTCCTTGCTGCACCCGGTGCTACCTCACGAATGGACGAGCACTTCCCCGAAGCTATGCTGT

GGGAACCCCACCGATGGGACGAGAACCCAAGTGAGAAGTACGCACATCTCGCACCAAAGCATGTCAAGGAGGGCGTCGC

CGAAGAGACTGAAGATTACGGCGATGGTCTCGTCAGCAAAGGCGCCGCATCACCATATCTGCCATTCGGTGCTGGCCGA

CATAGATGCATCGGCGAGCAATTCGCCTATGTCCAGCTCCAGACCATCACCTCGGAAGTGATTCGCGATTTCAAGCTCT

ACAATGTCGACGGCAGCGACAAAGTTGTCGGCACAGATTACAGTTCGTTGTTCAGCAGACCTCTCTCGCCAGCCGTCGT

GCGATGGGAGAGGAGAGAAAAGAAATAGATTCACAGTAAGGA

>E_22

AGATGGTGCAGATGATGGTTGGACCCTTGGACCCTCGCAATTGATGAGAAGCAGGGGTGTCCCGTTCCTGCATGACTAG

CGCCAAAAAGTGACGCGAATGCCGAAATGTTAAATCTCGTACGATAGCACCTGCCCATGGACCACTCGAACCACACTGA

AGGGTAATCATTCAAGATCTTGGTGATTTGGCCTAACCCCCTACACCAACATCAACATCACTGACTCCGCGCAATGGGA

CTCCTCCAGGACGCCGCGGCGCTTTTCGACGCGCAATTTGGCCAGATAGCGACATGGAAACTAGTCCCCCTCGGCTTCA

GCATCTTCTTCGCCGTATCCGTGTTGCTTAACGTGTTGCGCCAGCTGCTTTTCAGAAATCCAAACGAACCTCCGCTAGT

ATTCCACTACGTGCCCTTCATTGGCAGCACTATCTCCTATGGCATCGACCCCTACAAGTTCTTCTTCGCCTGCCGTCAA

AAATACGGAGATTGCTTCACTTTCATCCTCCTTGGCAAGAAGACCACCGTGGTGCTGGGGACTAAAGGCAACGACTTTA

TCTTGAATGGAAAGCTCAAGGACGTCAATGCCGAGGAGATCTATAGCCCACTTACTACGCCAGTATTCGGCACAGATGT

CGTCTACGATTGTCCCAATTCGAAGCTCATGGAGCAGAAGAAGGTGCGTGCACACAATAAGCTGGATTTATGACAATGC

TAACTTATACAGTTCGTCAAATATGGTCAGTGACTTGACTCCGCCGCGTCTGAGACAGCGCTCTCAACATGAGCTTCCA

CTTTGGAGCGATACACTTGCTAACCTGCGCCTCCAGGCCTCACCTCCTCCGCCCTCCAGTCCTACGTTAAATTGATCAC

CAAAGAGACCAAAGACTTCTTCTCCAAGGACAATCCAAGCAAGAAATTCGCATCCACCCACGGCACCGTCGACCTCCCG

CCTGCTATGGCTGAGCTTACTATCTACACCGCCAGCCGTTCGCTCCAGGGCAAAGAAGTCCGCGAAAAATTCGACTCCT

CCTTTGCCGACCTCTACCACGATCTCGACATGGGCTTCACTCCCATCAACTTCATGCTTCCATGGGCTCCACTGCCACA

GAATAGAGCACGCGATCGCGCACAGAAGAAGATGGCGGAAGTCTACACAGCGATCATCAAAGAGCGACGCGAAAAGGGC

GAGCCTACTTCGGGAGAGAAAGAGCAGGACATGATTTGGAATCTGATGCAATGTCAGTACAAGAATGGTCAAGCAATTC

CAGATAAGGAGATTGCGCACATGATGATTGCCCTTCTCATGGCTGGTCAACACTCGTCCTCGTCCACCTCATGCTGGAT

CCTTCTCCGACTGGCTTCGCGACCAGATATCCAGGATGAGCTCCTTCAAGAACAGAAGGATGTGCTTGGCGTCAACGCA

GATGGATCAATCAAGGAGTTAACGTACGCCGACATCTCGCGCCTTCCACTCCTCAATCAAGTTGTCAAGGAGACACTCC

GCCTTCATGCTCCCATCCATTCTATTCTGCGACAAGTCAAGTCTCCGATGCCACTCGAAGGTACACCATACGTTGTCCC

GACCACACACTCCCTCCTTGCTGCACCCGGTGCTACCTCACGAATGGACGAGCACTTCCCCGAAGCTATGCTGTGGGAA

CCCCACCGATGGGACGAGAACCCAAGTGAGAAGTACGCACATCTCGCACCAAAGCATGTCAAGGAGGGCGTCGCCGAAG

AGACTGAAGATTACGGCTATGGTCTCGTCAGCAAAGGCGCCGCATCACCATATCTGCCATTCGGTGCTGGCCGACATAG

ATGCATCGGCGAGCAATTCGCCTATGTCCAGCTCCAGACCATCACCTCGGAAGTGATTCGCGATTTCAAGCTCTACAAT

GTCGACGGCAGCGACAAAGTTGTCGGCACAGATTACAGTTCGTTGTTCAGCAGACCTCTCTCGCCAGCCGTCGTGCGAT

GGGAGAGGAGAGAAAAGAAATAGATCACAGTAAA

>GS_10-Consensus

CGAGATGGTGCAGATGATGGTTGGACCCTTGGACCCTCGCAATTGA

TGAGAAGCAGGGGTGTCCCGTTCCTGCATGACTAGCGCCAAAAAGTGACGCGAATGCCGA

AATGTTAAATCTCGTACGATAGCACCTGCCCATGGACCAC

TCGAACCACACTGAAGGGTAATCATTCAAGATCTTGGTGATTTGGCCTAACCCCC

TACACCAACATCAACATCACTGACTCCGCGCAATGGGACTCCTCCAGGACGCCGCGGCGC

TTTTCGACGCGCAATTTGGCCAGATAGCGACATGGAAACTAGTCCCCCTCGGCTTCAGCA

TCTTCTTCGCCGTATCCGTGTTGCTTAACGTGTTGCGCCAGCTGCTTTTCAGAAATCCAA

ACGAACCTCCGCTAGTATTCCACTACGTGCCCTTCATTGGCAGCACTATCTCCTATGGCA

TCGACCCCTACAAGTTCTTCTTCGCCTGCCGTCAAAAATACGGAGATTGCTTCACTTTCA

TCCTCCTTGGCAAGAAGACCACCGTGGTGCTGGGGACTAAAGGCAACGACTTTATCTTGA

ATGGAAAGCTCAAGGACGTCAATGCCGAGGAGATCTATAGCCCACTTACTACGCCAGTAT

TCGGCACAGATGTCGTCTACGATTGTCCCAATTCGAAGCTCATGGAGCAGAAGAAGGTGC

GTGCACACAATAAGCTGGATTTATGACAATGCTAACTTATACAGTTCGTCAAATATGGTC

AGTGACTTGACTCCGCCGCGTCTGAGACAGCGCTCTCAACATGAGCTTCCACTTTGGAGC

GATACACTTGCTAACCTGCGCCTCCAGGCCTCACCTCCTCCGCCCTCCAGTCCTACGTTA

AATTGATCACCAAAGAGACCAAAGACTTCTTCTCCAAGGACAATCCAAGCAAGAAATTCG

CATCCACCCACGGCACCGTCGACCTCCCGCCTGCTATGGCTGAGCTTACTATCTACACCG

CCAGCCGTTCGCTCCAGGGCAAAGAAGTCCGCGAAAAATTCGACTCCTCCTTTGCCGACC

TCTACCACGATCTCGACATGGGCTTCACTCCCATCAACTTCATGCTTCCATGGGCTCCAC

TGCCACAGAATAGAGCACGCGATCGCGCACAGAAGAAGATGGCGGAAGTCTACACAGCG

ATCATCAAAGAGCGACGCGAAAAGGGCGAGCCTACTTCGGGAGAGAAAGAGCAGGACATG

ATTTGGAATCTGATGCAATGTCAGTACAAGAATGGTCAAGCAATTCCAGATAAGGAGATT

GCGCACATGATGATTGCCCTTCTCATGGGTGGTCAACACTCGTCCTCGTCCACCTCATGC

TGGATCCTTCTCCGACTGGCTTCGCGACCAGATATCCAGGATGAGCTCCTTCAAGAACAG

AAGGATGTGCTTGGCGTCAACGCAGATGGATCAATCAAGGAGTTAACGTACGCCGACATC

TCGCGCCTTCCACTCCTCAATCAAGTTGTCAAGGAGACACTCCGCCTTCATGCTCCCATC

CATTCTATTCTGCGACAAGTCAAGTCTCCGATGCCACTCGAAGGTACACCATACGTTGTC

CCGACCACACACTCCCTCCTTGCTGCACCCGGTGCTACCTCACGAATGGACGAGCACTTC

CCCGAAGCTATGCTGTGGGAACCCCACCGATGGGACGAGAACCCAAGTGAGAAGTACGCA

CATCTCGCACCAAAGCATGTCAAGGAGGGCGTCGCCGAAGAGACTGAAGATTACGGCAAT

GGTCTCGTCAGCAAAGGCGCCGCATCACCATATCTGCCATTCGGTGCTGGCCGACATAGA

TGCATCGGCGAGCAATTCGCCTATGTCCAGCTCCAGACCATCACCTCGGAAGTGATTCGC

GATTTCAAGCTCTACAATGTCGACGGCAGCGACAAAGTTGTCGGCACAGATTACAGTTCG

TTGTTCAGCAGACCTCTCTCGCCAGCCGTCGTGCGATGGGAGAGGAGAGAAAGAAATA

GATTTCACAGTA

>RN_3-Consensus

CGAGATGGTGCAGATGATGGTTGGACCCTTG-GACCCTCGCAATTGA

TGAGAAGCAGGGGTGTCCCGTTCCTGCATGACTAGCGCCAAAAAGTGACGCGAATGCCGA

AATGTTAAATCTCGTACGATAGCACCTGCCCATGGACCAC

TCGAACCACACTGAAGGGTAATCATTCAAGATCTTGGTGATTTGGCCTAACCCCC

TACACCAACATCAACATCACTGACTCCGCGCAATGGGACTCCTCCAGGACGCCGCGGCGC

TTTTCGACGCGCAATTTGGCCAGATAGCGACATGGAAACTAGTCCCCCTCGGCTTCAGCA

TCTTCTTCGCCGTATCCGTGTTGCTTAACGTGTTGCGCCAGCTGCTTTTCAGAAATCCAA

ACGAACCTCCGCTAGTATTCCACTACGTGCCCTTCATTGGCAGCACTATCTCCTATGGCA

TCGACCCCTACAAGTTCTTCTTCGCCTGCCGTCAAAAATACGGAGATTGCTTCACTTTCA

TCCTCCTTGGCAAGAAGACCACCGTGGTGCTGGGGACTAAAGGCAACGACTTTATCTTGA

ATGGAAAGCTCAAGGACGTCAATGCCGAGGAGATCTATAGCCCACTTACTACGCCAGTAT

TCGGCACAGATGTCGTCTACGATTGTCCCAATTCGAAGCTCATGGAGCAGAAGAAGGTGC

GTGCACACAATAAGCTGGATTTATGACAATGCTAACTTATACAGTTCGTCAAATATGGTC

AGTGACTTGACTCCGCCGCGTCTGAGACAGCGCTCTCAACATGAGCTTCCACTTTGGAGC

GATACACTTGCTAACCTGCGCCTCCAGGCCTCACCTCCTCCGCCCTCCAGTCCTACGTTA

AATTGATCACCAAAGAGACCAAAGACTTCTTCTCCAAGGACAATCCAAGCAAGAAATTCG

CATCCACCCACGGCACCGTCGACCTCCCGCCTGCTATGGCTGAGCTTACTATCTACACCG

CCAGCCGTTCGCTCCAGGGCAAAGAAGTCCGCGAAAAATTCGACTCCTCCTTTGCCGACC

TCTACCACGATCTCGACATGGGCTTCACTCCCATCAACTTCATGCTTCCATGGGCTCCAC

TGCCACAGAA-TAGAGCACGCGATCGCGCACAGAAGAAGATGGCGGAAGTCTACACAGCG

ATCATCAAAGAGCGACGCGAAAAGGGCGAGCCTACTTCGGGAGAGAAAGAGCAGGACATG

ATTTGGAATCTGATGCAATGTCAGTACAAGAATGGTCAAGCAATTCCAGATAAGGAGATT

GCGCACATGATGATTGCCCTTCTCATGGGTGGTCAACACTCGTCCTCGTCCACCTCATGC

TGGATCCTTCTCCGACTGGCTTCGCGACCAGATATCCAGGATGAGCTCCTTCAAGAACAG

AAGGATGTGCTTGGCGTCAACGCAGATGGATCAATCAAGGAGTTAACGTACGCCGACATC

TCGCGCCTTCCACTCCTCAATCAAGTTGTCAAGGAGACACTCCGCCTTCATGCTCCCATC

CATTCTATTCTGCGACAAGTCAAGTCTCCGATGCCACTCGAAGGTACACCATACGTTGTC

CCGACCACACACTCCCTCCTTGCTGCACCCGGTGCTACCTCACGAATGGACGAGCACTTC

CCCGAAGCTATGCTGTGGGAACCCCACCGATGGGACGAGAACCCAAGTGAGAAGTACGCA

CATCTCGCACCAAAGCATGTCAAGGAGGGCGTCGCCGAAGAGACTGAAGATTACGGCCAT

GGTCTCGTCAGCAAAGGCGCCGCATCACCATATCTGCCATTCGGTGCTGGCCGACATAGA

TGCATCGGCGAGCAATTCGCCTATGTCCAGCTCCAGACCATCACCTCGGAAGTGATTCGC

GATTTCAAGCTCTACAATGTCGACGGCAGCGACAAAGTTGTCGGCACAGATTACAGTTCG

TTGTTCAGCAGACCTCTCTCGCCAGCCGTCGTGCGATGGGAGAGGAGAGAAAGAATA

GATTTCACAGTAAGA

>RN_5-Consensus

CGAGATGGTGCAGATGATGGTTGGACCCTTGGACCCTCGCAATTGA

TGAGAAGCAGGGGTGTCCCGTTCCTGCATGACTAGCGCCAAAAAGTGACGCGAATGCCGA

AATGTTAAATCTCGTACGATAGCACCTGCCCATGGACCAC

TCGAACCACACTGAAGGGTAATCATTCAAGATCTTGGTGATTTGGCCTAACCCCC

TACACCAACATCAACATCACTGACTCCGCGCAATGGGACTCCTCCAGGACGCCGCGGCGC

TTTTCGACGCGCAATTTGGCCAGATAGCGACATGGAAACTAGTCCCCCTCGGCTTCAGCA

TCTTCTTCGCCGTATCCGTGTTGCTTAACGTGTTGCGCCAGCTGCTTTTCAGAAATCCAA

ACGAACCTCCGCTAGTATTCCACTACGTGCCCTTCATTGGCAGCACTATCTCCTATGGCA

TCGACCCCTACAAGTTCTTCTTCGCCTGCCGTCAAAAATACGGAGATTGCTTCACTTTCA

TCCTCCTTGGCAAGAAGACCACCGTGGTGCTGGGGACTAAAGGCAACGACTTTATCTTGA

ATGGAAAGCTCAAGGACGTCAATGCCGAGGAGATCTATAGCCCACTTACTACGCCAGTAT

TCGGCACAGATGTCGTCTACGATTGTCCCAATTCGAAGCTCATGGAGCAGAAGAAGGTGC

GTGCACACAATAAGCTGGATTTATGACAATGCTAACTTATACAGTTCGTCAAATATGGTC

AGTGACTTGACTCCGCCGCGTCTGAGACAGCGCTCTCAACATGAGCTTCCACTTTGGAGC

GATACACTTGCTAACCTGCGCCTCCAGGCCTCACCTCCTCCGCCCTCCAGTCCTACGTTA

AATTGATCACCAAAGAGACCAAAGACTTCTTCTCCAAGGACAATCCAAGCAAGAAATTCG

CATCCACCCACGGCACCGTCGACCTCCCGCCTGCTATGGCTGAGCTTACTATCTACACCG

CCAGCCGTTCGCTCCAGGGCAAAGAAGTCCGCGAAAAATTCGACTCCTCCTTTGCCGACC

TCTACCACGATCTCGACATGGGCTTCACTCCCATCAACTTCATGCTTCCATGGGCTCCAC

TGCCACAGAATAGAGCACGCGATCGCGCACAGAAGAAGATGGCGGAAGTCTACACAGCG

ATCATCAAAGAGCGACGCGAAAAGGGCGAGCCTACTTCGGGAGAGAAAGAGCAGGACATG

ATTTGGAATCTGATGCAATGTCAGTACAAGAATGGTCAAGCAATTCCAGATAAGGAGATT

GCGCACATGATGATTGCCCTTCTCATGGGTGGTCAACACTCGTCCTCGTCCACCTCATGC

TGGATCCTTCTCCGACTGGCTTCGCGACCAGATATCCAGGATGAGCTCCTTCAAGAACAG

AAGGATGTGCTTGGCGTCAACGCAGATGGATCAATCAAGGAGTTAACGTACGCCGACATC

TCGCGCCTTCCACTCCTCAATCAAGTTGTCAAGGAGACACTCCGCCTTCATGCTCCCATC

CATTCTATTCTGCGACAAGTCAAGTCTCCGATGCCACTCGAAGGTACACCATACGTTGTC

CCGACCACACACTCCCTCCTTGCTGCACCCGGTGCTACCTCACGAATGGACGAGCACTTC

CCCGAAGCTATGCTGTGGGAACCCCACCGATGGGACGAGAACCCAAGTGAGAAGTACGCA

CATCTCGCACCAAAGCATGTCAAGGAGGGCGTCGCCGAAGAGACTGAAGATTACGGCCAT

GGTCTCGTCAGCAAAGGCGCCGCATCACCATATCTGCCATTCGGTGCTGGCCGACATAGA

TGCATCGGCGAGCAATTCGCCTATGTCCAGCTCCAGACCATCACCTCGGAAGTGATTCGC

GATTTCAAGCTCTACAATGTCGACGGCAGCGACAAAGTTGTCGGCACAGATTACAGTTCG

TTGTTCAGCAGACCTCTCTCGCCAGCCGTCGTGCGATGGGAGAGGAGAGAAAGAATA

GATTTCACAGTAAGAGTATGTTAATGCTAATCA

>RS_13-Consensus

TCGCAATTGA

TGAGAAGCAGGGGTGTCCCGTTCCTGCATGACTAGCGCCAAAAAGTGACGCGAATGCCGA

AATGTTAAATCTCGTACGATAGCACCTGCCCATGGACCAC

TCGAACCACACTGAAGGGTAATCATTCAAGATCTTGGTGATTTGGCCTAACCCCC

TACACCAACATCAACATCACTGACTCCGCGCAATGGGACTCCTCCAGGACGCCGCGGCGC

TTTTCGACGCGCAATTTGGCCAGATAGCGACATGGAAACTAGTCCCCCTCGGCTTCAGCA

TCTTCTTCGCCGTATCCGTGTTGCTTAACGTGTTGCGCCAGCTGCTTTTCAGAAATCCAA

ACGAACCTCCGCTAGTATTCCACTACGTGCCCTTCATTGGCAGCACTATCTCCTATGGCA

TCGACCCCTACAAGTTCTTCTTCGCCTGCCGTCAAAAATACGGAGATTGCTTCACTTTCA

TCCTCCTTGGCAAGAAGACCACCGTGGTGCTGGGGACTAAAGGCAACGACTTTATCTTGA

ATGGAAAGCTCAAGGACGTCAATGCCGAGGAGATCTATAGCCCACTTACTACGCCAGTAT

TCGGCACAGATGTCGTCTACGATTGTCCCAATTCGAAGCTCATGGAGCAGAAGAAGGTGC

GTGCACACAATAAGCTGGATTTATGACAATGCTAACTTATACAGTTCGTCAAATATGGTC

AGTGACTTGACTCCGCCGCGTCTGAGACAGCGCTCTCAACATGAGCTTCCACTTTGGAGC

GATACACTTGCTAACCTGCGCCTCCAGGCCTCACCTCCTCCGCCCTCCAGTCCTACGTTA

AATTGATCACCAAAGAGACCAAAGACTTCTTCTCCAAGGACAATCCAAGCAAGAAATTCG

CATCCACCCACGGCACCGTCGACCTCCCGCCTGCTATGGCTGAGCTTACTATCTACACCG

CCAGCCGTTCGCTCCAGGGCAAAGAAGTCCGCGAAAAATTCGACTCCTCCTTTGCCGACC

TCTACCACGATCTCGACATGGGCTTCACTCCCATCAACTTCATGCTTCCATGGGCTCCAC

TGCCACAGAA-TAGAGCACGCGATCGCGCACAGAAGAAGATGGCGGAAGTCTACACAGCG

ATCATCAAAGAGCGACGCGAAAAGGGCGAGCCTACTTCGGGAGAGAAAGAGCAGGACATG

ATTTGGAATCTGATGCAATGTCAGTACAAGAATGGTCAAGCAATTCCAGATAAGGAGATT

GCGCACATGATGATTGCCCTTCTCATGGGTGGTCAACACTCGTCCTCGTCCACCTCATGC

TGGATCCTTCTCCGACTGGCTTCGCGACCAGATATCCAGGATGAGCTCCTTCAAGAACAG

AAGGATGTGCTTGGCGTCAACGCAGATGGATCAATCAAGGAGTTAACGTACGCCGACATC

TCGCGCCTTCCACTCCTCAATCAAGTTGTCAAGGAGACACTCCGCCTTCATGCTCCCATC

CATTCTATTCTGCGACAAGTCAAGTCTCCGATGCCACTCGAAGGTACACCATACGTTGTC

CCGACCACACACTCCCTCCTTGCTGCACCCGGTGCTACCTCACGAATGGACGAGCACTTC

CCCGAAGCTATGCTGTGGGAACCCCACCGATGGGACGAGAACCCAAGTGAGAAGTACGCA

CATCTCGCACCAAAGCATGTCAAGGAGGGCGTCGCCGAAGAGACTGAAGATTACGGCAAT

GGTCTCGTCAGCAAAGGCGCCGCATCACCATATCTGCCATTCGGTGCTGGCCGACATAGA

TGCATCGGCGAGCAATTCGCCTATGTCCAGCTCCAGACCATCACCTCGGAAGTGATTCGC

GATTTCAAGCTCTACAATGTCGACGGCAGCGACAAAGTTGTCGGCACAGATTACAGTTCG

TTGTTCAGCAGACCTCTCTCGCCAGCCGTCGTGCGATGGGAGAGGAGAGAAAAGAAATA

GATTTCACAGTAGAGTATG

>SaR_2-Consensus

GAGATGGTGCAGATGATGGTTGGACCCTTG-GACCCTCGCAATTGA

TGAGAAGCAGGGGTGTCCCGTTCCTGCATGACTAGCGCCAAAAAGTGACGCGAATGCCGA

AATGTTAAATCTCGTACGATAGCACCTGCCCATGGACCAC

TCGAACCACACTGAAGGGTAATCATTCAAGATCTTGGTGATTTGGCCTAACCCCC

TACACCAACATCAACATCACTGACTCCGCGCAATGGGACTCCTCCAGGACGCCGCGGCGC

TTTTCGACGCGCAATTTGGCCAGATAGCGACATGGAAACTAGTCCCCCTCGGCTTCAGCA

TCTTCTTCGCCGTATCCGTGTTGCTTAACGTGTTGCGCCAGCTGCTTTTCAGAAATCCAA

ACGAACCTCCGCTAGTATTCCACTACGTGCCCTTCATTGGCAGCACTATCTCCTATGGCA

TCGACCCCTACAAGTTCTTCTTCGCCTGCCGTCAAAAATACGGAGATTGCTTCACTTTCA

TCCTCCTTGGCAAGAAGACCACCGTGGTGCTGGGGACTAAAGGCAACGACTTTATCTTGA

ATGGAAAGCTCAAGGACGTCAATGCCGAGGAGATCTATAGCCCACTTACTACGCCAGTAT

TCGGCACAGATGTCGTCTACGATTGTCCCAATTCGAAGCTCATGGAGCAGAAGAAGGTGC

GTGCACACAATAAGCTGGATTTATGACAATGCTAACTTATACAGTTCGTCAAATATGGTC

AGTGACTTGACTCCGCCGCGTCTGAGACAGCGCTCTCAACATGAGCTTCCACTTTGGAGC

GATACACTTGCTAACCTGCGCCTCCAGGCCTCACCTCCTCCGCCCTCCAGTCCTACGTTA

AATTGATCACCAAAGAGACCAAAGACTTCTTCTCCAAGGACAATCCAAGCAAGAAATTCG

CATCCACCCACGGCACCGTCGACCTCCCGCCTGCTATGGCTGAGCTTACTATCTACACCG

CCAGCCGTTCGCTCCAGGGCAAAGAAGTCCGCGAAAAATTCGACTCCTCCTTTGCCGACC

TCTACCACGATCTCGACATGGGCTTCACTCCCATCAACTTCATGCTTCCATGGGCTCCAC

TGCCACAGAA-TAGAGCACGCGATCGCGCACAGAAGAAGATGGCGGAAGTCTACACAGCG

ATCATCAAAGAGCGACGCGAAAAGGGCGAGCCTACTTCGGGAGAGAAAGAGCAGGACATG

ATTTGGAATCTGATGCAATGTCAGTACAAGAATGGTCAAGCAATTCCAGATAAGGAGATT

GCGCACATGATGATTGCCCTTCTCATGGGTGGTCAACACTCGTCCTCGTCCACCTCATGC

TGGATCCTTCTCCGACTGGCTTCGCGACCAGATATCCAGGATGAGCTCCTTCAAGAACAG

AAGGATGTGCTTGGCGTCAACGCAGATGGATCAATCAAGGAGTTAACGTACGCCGACATC

TCGCGCCTTCCACTCCTCAATCAAGTTGTCAAGGAGACACTCCGCCTTCATGCTCCCATC

CATTCTATTCTGCGACAAGTCAAGTCTCCGATGCCACTCGAAGGTACACCATACGTTGTC

CCGACCACACACTCCCTCCTTGCTGCACCCGGTGCTACCTCACGAATGGACGAGCACTTC

CCCGAAGCTATGCTGTGGGAACCCCACCGATGGGACGAGAACCCAAGTGAGAAGTACGCA

CATCTCGCACCAAAGCATGTCAAGGAGGGCGTCGCCGAAGAGACTGAAGATGACGGCTAT

GGTCTCGTCAGCAAAGGCGCCGCATCACCATATCTGCCATTCGGTGCTGGCCGACATAGA

TGCATCGGCGAGCAATTCGCCTATGTCCAGCTCCAGACCATCACCTCGGAAGTGATTCGC

GATTTCAAGCTCTACAATGTCGACGGCAGCGACAAAGTTGTCGGCACAGATTACAGTTCG

TTGTTCAGCAGACCTCTCTCGCCAGCCGTCGTGCGATGGGAGAGGAGAGAAAGAAATA

GATTTCACAGTAAGAG

>SaR_5-Consensus

GGTGCAGATGATGGTTGGACCCTTG-GACCCTCGCAATTGA

TGAGAAGCAGGGGTGTCCCGTTCCTGCATGACTAGCGCCAAAAAGTGACGCGAATGCCGA

AATGTTAAATCTCGTACGATAGCACCTGCCCATGGACCAC

TCGAACCACACTGAAGGGTAATCATTCAAGATCTTGGTGATTTGGCCTAACCCCC

TACACCAACATCAACATCACTGACTCCGCGCAATGGGACTCCTCCAGGACGCCGCGGCGC

TTTTCGACGCGCAATTTGGCCAGATAGCGACATGGAAACTAGTCCCCCTCGGCTTCAGCA

TCTTCTTCGCCGTATCCGTGTTGCTTAACGTGTTGCGCCAGCTGCTTTTCAGAAATCCAA

ACGAACCTCCGCTAGTATTCCACTACGTGCCCTTCATTGGCAGCACTATCTCCTATGGCA

TCGACCCCTACAAGTTCTTCTTCGCCTGCCGTCAAAAATACGGAGATTGCTTCACTTTCA

TCCTCCTTGGCAAGAAGACCACCGTGGTGCTGGGGACTAAAGGCAACGACTTTATCTTGA

ATGGAAAGCTCAAGGACGTCAATGCCGAGGAGATCTATAGCCCACTTACTACGCCAGTAT

TCGGCACAGATGTCGTCTACGATTGTCCCAATTCGAAGCTCATGGAGCAGAAGAAGGTGC

GTGCACACAATAAGCTGGATTTATGACAATGCTAACTTATACAGTTCGTCAAATATGGTC

AGTGACTTGACTCCGCCGCGTCTGAGACAGCGCTCTCAACATGAGCTTCCACTTTGGAGC

GATACACTTGCTAACCTGCGCCTCCAGGCCTCACCTCCTCCGCCCTCCAGTCCTACGTTA

AATTGATCACCAAAGAGACCAAAGACTTCTTCTCCAAGGACAATCCAAGCAAGAAATTCG

CATCCACCCACGGCACCGTCGACCTCCCGCCTGCTATGGCTGAGCTTACTATCTACACCG

CCAGCCGTTCGCTCCAGGGCAAAGAAGTCCGCGAAAAATTCGACTCCTCCTTTGCCGACC

TCTACCACGATCTCGACATGGGCTTCACTCCCATCAACTTCATGCTTCCATGGGCTCCAC

TGCCACAGAA-TAGAGCACGCGATCGCGCACAGAAGAAGATGGCGGAAGTCTACACAGCG

ATCATCAAAGAGCGACGCGAAAAGGGCGAGCCTACTTCGGGAGAGAAAGAGCAGGACATG

ATTTGGAATCTGATGCAATGTCAGTACAAGAATGGTCAAGCAATTCCAGATAAGGAGATT

GCGCACATGATGATTGCCCTTCTCATGGGTGGTCAACACTCGTCCTCGTCCACCTCATGC

TGGATCCTTCTCCGACTGGCTTCGCGACCAGATATCCAGGATGAGCTCCTTCAAGAACAG

AAGGATGTGCTTGGCGTCAACGCAGATGGATCAATCAAGGAGTTAACGTACGCCGACATC

TCGCGCCTTCCACTCCTCAATCAAGTTGTCAAGGAGACACTCCGCCTTCATGCTCCCATC

CATTCTATTCTGCGACAAGTCAAGTCTCCGATGCCACTCGAAGGTACACCATACGTTGTC

CCGACCACACACTCCCTCCTTGCTGCACCCGGTGCTACCTCACGAATGGACGAGCACTTC

CCCGAAGCTATGCTGTGGGAACCCCACCGATGGGACGAGAACCCAAGTGAGAAGTACGCA

CATCTCGCACCAAAGCATGTCAAGGAGGGCGTCGCCGAAGAGACTGAAGATTACGGCAAT

GGTCTCGTCAGCAAAGGCGCCGCATCACCATATCTGCCATTCGGTGCTGGCCGACATAGA

TGCATCGGCGAGCAATTCGCCTATGTCCAGCTCCAGACCATCACCTCGGAAGTGATTCGC

GATTTCAAGCTCTACAATGTCGACGGCAGCGACAAAGTTGTCGGCACAGATTACAGTTCG

TTGTTCAGCAGACCTCTCTCGCCAGCCGTCGTGCGATGGGAGAGGAGAGAAAGAAATA

GATTTCACAGTA

>X845-Consensus

GCAGGTGCAAGGTGCGGCGTGATGGTTGGACCCTTGGACCCTCGCAATTGA

TGAGAAGCAGGGGTGTCCCGTTCCTGCATGACTAGCGCCAAAAAGTGACGCGACTGTCGA

AATGTTAAATCTCGTACGATTGCACCTGCCATGGACCAC

TCGAACAACACTGAAGGGTAATCATTCAAGACCTTGGTGATTTGGCCTAACCCCC

GACACGAACATCAACATCACTGACACCGCGCAATGGGGCTCCTCCAAGACGCTGCGGCGC

TTTTCGACGCGCAATTTGGCCAGATAGCGACATGGAAACTTGTCCCCCTCGGCTTCAGCA

TCTTCTTCGCCGTATCCGTGTTGCTTAACGTGTTGCGCCAGCTGCTCTTCAGAAATCCAA

ACGAACCTCCGCTAGTATTCCACTTCGTGCCCTTCATTGGCAGCACTATCTCCTATGGCA

TCGACCCCTACAAGTTCTTCTTCGCCTGCCGTCAAAAATACGGAGATTGCTTCACTTTCA

TCCTCCTTGGCAAGAAGACCACCGTGGTGCTGGGGACTAAAGGCAACGACTTTATCTTGA

ATGGAAAGCTCAAGGACGTCAATGCCGAGGAGATCTATAGCCCACTTACTACGCCAGTAT

TCGGCACAGATGTCGTCTACGATTGTCCCAATTCGAAGCTCATGGAGCAGAAGAAGGTGC

GTGCACACAATAAGCTGGATTTATGACAATGCTAACTTATACAGTTCGTCAAATATGGTC

AGTGACTTGACTCCGCCGCGTCTGAGACAGCGCTCTCAACATGAGCTTCCACTTTGGAGC

GATACACTTGCTAACCTGCGCCTCCAGGCCTCACCTCCTCCGCCCTCCAGTCCTACGTTA

AATTGATCACCAAAGAGACCAAAGACTTCTTCTCCAAGGACAATCCAAGCAAGAAATTCG

CATCCACCCACGGCACCGTCGACCTCCCGCCTGCTATGGCTGAGCTTACTATCTACACCG

CCAGCCGTTCGCTCCAGGGCAAAGAAGTCCGCGAAAAATTCGACTCCTCCTTTGCCGACC

TCTACCACGATCTCGACATGGGCTTCACTCCCATCAACTTCATGCTTCCATGGGCTCCAC

TGCCACAGAA-TAGAGCACGCGATCGCGCACAGAAGAAGATGGCGGAAGTCTACACAGCG

ATCATCAAAGAGCGACGCGAAAAGGGCGAGCCTACTTCGGGAGAGAAAGAGCAGGACATG

ATTTGGAATCTGATGCAATGTCAGTACAAGAATGGTCAAGCAATTCCAGATAAGGAGATT

GCGCACATGATGATTGCCCTTCTCATGGCTGGTCAACACTCGTCCTCGTCCACCTCATGC

TGGATCCTTCTCCGACTGGCTTCGCGACCAGATATCCAGGATGAGCTCCTTCAAGAACAG

AAGGATGTGCTTGGCGTCAACGCAGATGGATCAATCAAGGAGTTAACGTACGCCGACATC

TCGCGCCTTCCACTCCTCAATCAAGTTGTCAAGGAGACACTCCGCCTTCATGCTCCCATC

CATTCTATTCTGCGACAAGTCAAGTCTCCGATGCCACTCGAAGGTACACCATACGTTGTC

CCGACCACACACTCCCTCCTTGCTGCACCCGGTGCTACCTCACGAATGGACGAGCACTTC

CCCGAAGCTATGCTGTGGGAACCCCACCGATGGGACGAGAACCCAAGTGAGAAGTACGCA

CATCTCGCACCAAAGCATGTCAAGGAGGGCGTCGCCGAAGAGACTGAAGATTACGGCTAT

GGTCTCGTCAGCAAAGGCGCCGCATCACCATATCTGCCATTCGGTGCTGGCCGACATAGA

TGCATCGGCGAGCAATTCGCCTATGTCCAGCTCCAGACCATCACCTCGGAAGTGATTCGC

GATTTCAAGCTCTACAATGTCGACGGCAGCGACAAAGTTGTCGGCACAGATTACAGTTCG

TTGTTCAGCAGACCTCTCTCGCCAGCCGTCGTGCGAT-GGGAGAGGAGAGAAAAGAAATA

GATTTCACAGTAAGA

>X846-Consensus

CGAGATGGCGCAGGTGCAAGGTGCGGCGTGATGGTTGGACCCTTGGACCCTCGCAATTGA

TGAGAAGCAGGGGTGTCCCGTTCCTGCATGACTAGCGCCAAAAAGTGACGCGACTGCCGA

AATGTTAAATCTCGTACGATAGCACCTGCCCATGGACCAC

TCGAACATCACTGAAGGGTAATCATTCAAGATCTTGGTGATTTGGCCTAACCCCC

TACACCAACATCAACATCACTGACTCCGCGCAATGGGGCTCCTCCAGGACGCTGCGGCGC

TTTTCGACGCGCAATTTGGCCAGATAGCGACATGGAAACTTGTCCCCCTCGGCTTCAGCA

TCTTCTTCGCCGTATCCGTGTTGCTTAACGTGTTGCGCCAGCTGCTCTTCAGAAATCCAA

ACGAACCTCCGCTAGTATTCCACTACGTGCCCTTCATTGGCAGCACTATCTCCTATGGCA

TCGACCCCTACAAGTTCTTCTTCGCCTGCCGTCAAAAATACGGAGATTGCTTCACTTTCA

TCCTCCTTGGCAAGAAGACCACCGTGGTGCTGGGGACTAAAGGCAACGACTTTATCTTGA

ATGGAAAGCTCAAGGACCTCAATGCCGAGGAGATCTATAGCCCACTTACTACCCCAGTAT

TCGGCACAGATGTCGTCTACGATTGTCCCAATTCGAAGCTCATGGAGCAGAAGAAGGTGC

GTGCACACAATAAGCTGGGTTTATGACAATGCTAACTTATACAGTTCGTCAAATATGGTC

AGTGACTTGACTCCGCCGCGTCTGAGACAGCGCTCTGAACATGAGCTTCCACTTTGGAGC

GATACACTTGCTAACCTCCGCCTCCAGGCCTCACCTCCTCCGCCCTCCAGTCCTACGTTA

AATTGATCACCAAAGAGACCAAAGACTTCTTCTCCAAGGACAATCCAAGCAAGAAATTCG

CATCCACCCACGGCACCGTCGACCTCCCGCCTGCTATGGCTGAGCTTACTATCTACACCG

CCAGCCGTTCGCTCCAGGGCAAAGAAGTCCGCGAAAAATTCGACTCCTCCTTTGCCGACC

TCTACCACGATCTCGACATGGGCTTCACTCCCATCAACTTCATGCTTCCATGGGCTCCAC

TGCCACAGAA-TAGAGCACGCGATCGCGCACAGAAGAAGATGGCGGAAGTCTACACAGCG

ATCATCAAAGAGCGACGCGAAAAGGGCGAGCCTACTTCGGGAGAGAAAGAGCAGGACATG

ATTTGGAATCTGATGCAATGTCAGTACAAGAATGGTCAAGCAATTCCAGATAAGGAGATT

GCGCACATGATGATTGCCCTTCTCATGGCTGGTCAACACTCGTCCTCGTCCACCTCATGC

TGGATCCTTCTCCGACTGGCTTCGCGACCAGATATCCAGGATGAGCTCCTTCAAGAACAG

AAGGATGTGCTTGGCGTCAACGCAGATGGATCAATCAAGGAGTTAACGTACGCCGACATC

TCGCGCCTTCCACTCCTCAATCAAGTTGTCAAGGAGACACTCCGCCTTCATGCTCCCATC

CATTCTATTCTGCGACAAGTCAAGTCTCCGATGCCACTCGAAGGTACACCATACGTTGTC

CCGACCACACACTCCCTCCTTGCTGCACCCGGTGCTACCTCACGAATGGACGAGCACTTC

CCCGAAGCTATGCTGTGGGAACCCCACCGATGGGACGAGAACCCAAGTGAGAAGTACGCA

CATCTCGCACCAAAGCATGTCAAGGAGGGCGTCGCCGAAGAGACTGAAGATTACGGCTAT

GGTCTCGTCAGCAAAGGCGCCGCATCACCATATCTGCCATTCGGTGCTGGCCGACATAGA

TGCATCGGCGAGCAATTCGCCTATGTCCAGCTCCAGACCATCACCTCGGAAGTGATTCGC

GATTTCAAGCTCTACAATGTCGACGGCAGCGACAAAGTTGTCGGCACAGATTACAGTTCG

TTGTTCAGCAGACCTCTCTCGCCAGCCGTCGTGCGATGGGAGAGGAGAGAAAGAAATA

GATTTCACAGTAAGA

>x847_Consensus

GGTGCAGATGCAAGGTGCGGCGTGATGGTTGGACCCTTGGACCCTCGCAATTGA

TGAGAAGCAGGGGTGTCCCGTTCCTGCATGACTAGCGCCAAAAAGTGACGCGACTGCCGA

AATGTTAAATCTCGTACGATAGCACCTGCCCATGGACCAC

TCGAACCACACTGAAGGGTAATCATTCAAGATCTTGGTGATTTGGCCTAACTTCC

TACACCAACATCAACATCACTGACTCCGCGCAATGGGGCTCCTCCAGGACGCCGCGGCGC

TTTTCGACGCGCAATTTGGCCAGACAGCGACATGGAAACTAGTCCCCCTCGGCTTCAGCA

TCTTCTTCGCCGTATCCGTGTTGCTTAACGTGTTGCGCCAGCTGCTCTTCAGAAATCCGA

ACGAACCTCCGCTAGTATTCCACTACGTGCCCTTCATTGGCAGCACTATCTCCTATGGCA

TCGACCCCTACAAGTTCTTCTTCGCCTGCCGTCAAAAATACGGAGATTGCTTCACTTTCA

TCCTCCTTGGCAAGAAGACCACCGTGGTGCTGGGGACTAAAGGCAACGACTTCATCTTGA

ATGGAAAGCTCAAGGACGTCAATGCCGAGGAGATCTATAGCCCACTTACTACGCCAGTAT

TCGGCACAGATGTCGTCTACGATTGTCCCAATTCGAAGCTCATGGAGCAGAAGAAGGTGC

GTGCACACAATAAGCTGGATTTATGACAATGCTAACTTATACAGTTCGTCAAATATGGTC

AGTGACTTGACTCCGCCGCGTCTGAGACAGCGATCTGAACATGAGCTTCCACTTTGGAGC

GATACACTTGCTAACCTCCGCCTCCAGGCCTCACCTCCTCCGCCCTCCAGTCCTACGTTA

AATTGATCACCAAAGAGACCAGAGACTTCTTCTCCAAGGACAATCCAAGCAAGAAATTCG

CATCCACCCACGGCACCGTCGACCTCCCGCCTGCTATGGCTGAGCTTACTATCTACACCG

CCAGCCGTTCGCTCCAGGGCAAAGAAGTCCGCGAAAAATTCGACTCCTCCTTTGCCGACC

TCTACCACGATCTCGACATGGGCTTCACTCCCATCAACTTCATGCTTCCATGGGCTCCAC

TGCCACAGAATAGAGCACGCGATCGCGCACAGAAGAAGATGGCGGAAGTCTACACAGCG

ATCATCAAAGAGCGACGCGAAAAGGGCGAGCCTACTTCGGGAGAGAAAGAGCAGGACATG

ATTTGGAATCTGATGCAATGTCAGTACAAGAATGGTCAAGCAATTCCAGATAAGGAGATT

GCGCACATGATGATTGCCCTTCTCATGGCTGGTCAACACTCGTCTTCGTCCACCTCATGC

TGGATCCTTCTCCGACTGGCTTCGCGACCAGATATCCAGGATGAGCTCCTTCAAGAACAG

AAGGATGTGCTTGGCGTCAACGCAGATGGATCAATCAAGGAGTTAACGTACGCCGACATC

TCGCGCCTTCCACTCCTCAATCAAGTTGTCAAGGAGACACTCCGCCTTCATGCTCCCATC

CATTCTATTCTGCGACAAGTCAAGTCTCCGATGCCACTCGAAGGTACACCATACGTTGTC

CCGACCACACACTCCCTCCTTGCTGCACCCGGTGCTACCTCACGAATGGACGAGCACTTC

CCCGAAGCTATGCTGTGGGAACCCCACCGATGGGACGAGAACCCAAGTGAGAAGTACGCA

CATCTCTCACCAAAGCATGTCAAGGAGGGCGTCGCCGAAGAGACTGAAGATTACGGCTAT

GGTCTCGTCAGCAAAGGCGCCGCATCACCATATCTGCCATTCGGTGCTGGCCGACATAGA

TGCATCGGCGAGCAATTCGCCTATGTCCAGCTCCAGACCATCACCTCAGAAGTGATTCGC

GATTTCAAGCTCTACAATGTCGACGGCAGCGACAAAGTTGTCGGCACAGATTACAGTTCG

TTGTTCAGCAGACCTCTCTCGCCAGCCGTCGTGCGATGGGAGAGGAGAGAAAGAAATA

GATTTCACAGTAGAGTAT

>x849_Consensus

TTGGACCCTTGGACCCTCGCAATTGA

TGAGAAGCAGGGGTGTCCCGTTCCTGCATGACTAGCGCCAAAAAGTGACGCGACTGCCGA

AATGTTAAATCTCGTACGATAGCACCTGCCCATGGACCAC

TCGAACATCACTGAAGGGTAATCATTCAAGATCTTGGTGATTTGGCCTAACCCCC

TACACCAACATCAACATCACTGACTCCGCGCAATGGGGCTCCTCCAGGACGCCGCGGCGC

TTTTCGACGCGCAATTTGGCCAGACAGCGACATGGAAACTAGTCCCCCTCGGCTTCAGCA

TCTTCTTCGCCGTATCCGTGTTGCTTAACGTGTTGCGCCAGCTGCTCTTCAGAAATCCAA

ACGAACCTCCGCTAGTATTCCACTACGTGCCCTTCATTGGCAGCACTATCTCCTATGGCA

TCGACCCCTACAAGTTCTTCTTCGCCTGCCGTCAAAAATACGGAGATTGCTTCACTTTCA

TCCTCCTTGGCAAGAAGACCACCGTGGTGCTGGGGACTAAAGGCAACGACTTTATCTTGA

ATGGAAAGCTCAAGGACGTCAATGCCGAGGAGATCTATAGCCCACTTACTACGCCAGTAT

TCGGCACAGATGTCGTCTACGATTGTCCCAATTCGAAGCTCATGGAGCAGAAGAAGGTGC

GTGCACACAATAAGCTGGATTTATGACAATGCTAACTTATACAGTTCGTCAAATATGGTC

AGTGACTTGACTCCGCCGCGTCTGAGACAGCGCTCTCAACATGAGCTTCCACTTTGGAGC

GATACACTTGCTAACCTGCGCCTCCAGGCCTCACCTCCTCCGCCCTCCAGTCCTACGTTA

AATTGATCACCAAAGAGACCAAAGACTTCTTCTCCAAGGACAATCCAAGCAAGAAATTCG

CATCCACCCACGGCACCGTCGACCTCCCGCCTGCTATGGCTGAGCTTACTATCTACACCG

CCAGCCGTTCGCTCCAGGGCAAAGAAGTCCGCGAAAAATTCGACTCCTCCTTTGCCGACC

TCTACCACGATCTCGACATGGGCTTCACTCCCATCAACTTCATGCTTCCATGGGCTCCAC

TGCCACAGAATAGAGCACGCGATCGCGCACAGAAGAAGATGGCGGAAGTCTACACAGCG

ATCATCAAAGAGCGACGCGAAAAGGGCGAGCCTACTTCGGGAGAGAAAGAGCAGGACATG

ATTTGGAATCTGATGCAATGTCAGTACAAGAATGGTCAAGCAATTCCAGATAAGGAGATT

GCGCACATGATGATTGCCCTTCTCATGGCTGGTCAACACTCGTCCTCGTCCACCTCATGC

TGGATCCTTCTCCGACTGGCTTCGCGACCAGATATCCAGGATGAGCTCCTTCAAGAACAG

AAGGATGTGCTTGGCGTCAACGCAGATGGATCAATCAAGGAGTTAACGTACGCCGACATC

TCGCGCCTTCCACTCCTCAATCAAGTTGTCAAGGAGACACTCCGCCTTCATGCTCCCATC

CATTCTATTCTGCGACAAGTCAAGTCTCCGATGCCACTCGAAGGTACACCATACGTTGTC

CCGACCACACACTCCCTCCTTGCTGCACCCGGTGCTACCTCACGAATGGACGAGCACTTC

CCCGAAGCTATGCTGTGGGAACCCCACCGATGGGACGAGAACCCAAGTGAGAAGTACGCA

CATCTCGCACCAAAGCATGTCAAGGAGGGCGTCGCCGAAGAGACTGAAGATTACGGCTAT

GGTCTCGTCAGCAAAGGCGCCGCATCACCATATCTGCCATTCGGTGCTGGCCGACATAGA

TGCATCGGCGAGCAATTCGCCTATGTCCAGCTCCAGACCATCACCTCGGAAGTGATTCGC

GATTTCAAGCTCTACAATGTCGACGGCAGCGACAAAGTTGTCGGCACAGATTACAGTTCG

TTGTTCAGCAGACCTCTCTCGCCAGCCGTCGTGCGATGGGAGAGGAGAGAAAAGAAATA

GATTTCACAGTAA

>ZTSC_77_Consensus

ATGGTGCAGATGATGGTTGGACCCTTG-GACCCTCGCAATTGA

TGAGAAGCAGGGGTGTCCCGTTCCTGCATGACTAGCGCCAAAAAGTGACGCGAATGCCGA

AATGTTAAATCTCGTACGATAGCACCTGCCCATGGACCACTCGAACCACACTGAAGGGTAATCATTCAAGATCTTGGTGATTTGGCCTAACCCCC

TACACCAACATCAACATCACTGACTCCGCGCAATGGGACTCCTCCAGGACGCCGCGGCGC

TTTTCGACGCGCAATTTGGCCAGATAGCGACATGGAAACTAGTCCCCCTCGGCTTCAGCA

TCTTCTTCGCCGTATCCGTGTTGCTTAACGTGTTGCGCCAGCTGCTTTTCAGAAATCCAA

ACGAACCTCCGCTAGTATTCCACTACGTGCCCTTCATTGGCAGCACTATCTCCTATGGCA

TCGACCCCTACAAGTTCTTCTTCGCCTGCCGTCAAAAATACGGAGATTGCTTCACTTTCA

TCCTCCTTGGCAAGAAGACCACCGTGGTGCTGGGGACTAAAGGCAACGACTTTATCTTGA

ATGGAAAGCTCAAGGACGTCAATGCCGAGGAGATCTATAGCCCACTTACTACGCCAGTAT

TCGGCACAGATGTCGTCTACGATTGTCCCAATTCGAAGCTCATGGAGCAGAAGAAGGTGC

GTGCACACAATAAGCTGGATTTATGACAATGCTAACTTATACAGTTCGTCAAATATGGTC

AGTGACTTGACTCCGCCGCGTCTGAGACAGCGCTCTCAACATGAGCTTCCACTTTGGAGC

GATACACTTGCTAACCTGCGCCTCCAGGCCTCACCTCCTCCGCCCTCCAGTCCTACGTTA

AATTGATCACCAAAGAGACCAAAGACTTCTTCTCCAAGGACAATCCAAGCAAGAAATTCG

CATCCACCCACGGCACCGTCGACCTCCCGCCTGCTATGGCTGAGCTTACTATCTACACCG

CCAGCCGTTCGCTCCAGGGCAAAGAAGTCCGCGAAAAATTCGACTCCTCCTTTGCCGACC

TCTACCACGATCTCGACATGGGCTTCACTCCCATCAACTTCATGCTTCCATGGGCTCCAC

TGCCACAGAATAGAGCACGCGATCGCGCACAGAAGAAGATGGCGGAAGTCTACACAGCG

ATCATCAAAGAGCGACGCGAAAAGGGCGAGCCTACTTCGGGAGAGAAAGAGCAGGACATG

ATTTGGAATCTGATGCAATGTCAGTACAAGAATGGTCAAGCAATTCCAGATAAGGAGATT

GCGCACATGATGATTGCCCTTCTCATGGGTGGTCAACACTCGTCCTCGTCCACCTCATGC

TGGATCCTTCTCCGACTGGCTTCGCGACCAGATATCCAGGATGAGCTCCTTCAAGAACAG

AAGGATGTGCTTGGCGTCAACGCAGATGGATCAATCAAGGAGTTAACGTACGCCGACATC

TCGCGCCTTCCACTCCTCAATCAAGTTGTCAAGGAGACACTCCGCCTTCATGCTCCCATC

CATTCTATTCTGCGACAAGTCAAGTCTCCGATGCCACTCGAAGGTACACCATACGTTGTC

CCGACCACACACTCCCTCCTTGCTGCACCCGGTGCTACCTCACGAATGGACGAGCACTTC

CCCGAAGCTATGCTGTGGGAACCCCACCGATGGGACGAGAACCCAAGTGAGAAGTACGCA

CATCTCGCACCAAAGCATGTCAAGGAGGGCGTCGCCGAAGAGACTGAAGATTACGGCTCT

GGTCTCGTCAGCAAAGGCGCCGCATCACCATATCTGCCATTCGGTGCTGGCCGACATAGA

TGCATCGGCGAGCAATTCGCCTATGTCCAGCTCCAGACCATCACCTCGGAAGTGATTCGC

GATTTCAAGCTCTACAATGTCGACGGCAGCGACAAAGTTGTCGGCACAGATTACAGTTCG

TTGTTCAGCAGACCTCTCTCGCCAGCCGTCGTGCGATGGGAGAGGAGAGAAAAGAAATA

GATTTCACAGTA

>ZTSC_79_Consensus

TGGTGCAGATGATGGTTGGACCCTTGGACCCTCGCAATTG

ATGAGAAGCAGGGGTGTGCGGTCCTGCATGACTAGCGCCAAAAAGTGACGCGAATGCCGA

AATGTTAAATCTCGTACGATAGCACCTGCCCATGGACCAC

TCGAACCACACTGAAGGGTAATCATTCAAGATCTTGGTGATTTGGCCTAACCCCC

TACACCAACATCAACATCACTGACTCCGCGCAATGGGACTCCTCCAGGACGCCGCGGCGC

TTTTCGACGCGCAATTTGGCCAGATAGCGACATGGAAACTAGTCCCCCTCGGCTTCAGCA

TCTTCTTCGCCGTATCCGTGTTGCTTAACGTGTTGCGCCAGCTGCTTTTCAGAAATCCAA

ACGAACCTCCGCTAGTATTCCACTACGTGCCCTTCATTGGCAGCACTATCTCCTATGGCA

TCGACCCCTACAAGTTCTTCTTCGCCTGCCGTCAAAAATACGGAGATTGCTTCACTTTCA

TCCTCCTTGGCAAGAAGACCACCGTGGTGCTGGGGACTAAAGGCAACGACTTTATCTTGA

ATGGAAAGCTCAAGGACGTCAATGCCGAGGAGATCTATAGCCCACTTACTACGCCAGTAT

TCGGCACAGATGTCGTCTACGATTGTCCCAATTCGAAGCTCATGGAGCAGAAGAAGGTGC

GTGCACACAATAAGCTGGATTTATGACAATGCTAACTTATACAGTTCGTCAAATATGGTC

AGTGACTTGACTCCGCCGCGTCTGAGACAGCGCTCTCAACATGAGCTTCCACTTTGGAGC

GATACACTTGCTAACCTGCGCCTCCAGGCCTCACCTCCTCCGCCCTCCAGTCCTACGTTA

AATTGATCACCAAAGAGACCAAAGACTTCTTCTCCAAGGACAATCCAAGCAAGAAATTCG

CATCCACCCACGGCACCGTCGACCTCCCGCCTGCTATGGCTGAGCTTACTATCTACACCG

CCAGCCGTTCGCTCCAGGGCAAAGAAGTCCGCGAAAAATTCGACTCCTCCTTTGCCGACC

TCTACCACGATCTCGACATGGGCTTCACTCCCATCAACTTCATGCTTCCATGGGCTCCAC

TGCCACAGAATAGAGCACGCGATCGCGCACAGAAGAAGATGGCGGAAGTCTACACAGCG

ATCATCAAAGAGCGACGCGAAAAGGGCGAGCCTACTTCGGGAGAGAAAGAGCAGGACATG

ATTTGGAATCTGATGCAATGTCAGTACAAGAATGGTCAAGCAATTCCAGATAAGGAGATT

GCGCACATGATGATTGCCCTTCTCATGGCTGGTCAACACTCGTCCTCGTCCACCTCATGC

TGGATCCTTCTCCGACTGGCTTCGCGACCAGATATCCAGGATGAGCTCCTTCAAGAACAG

AAGGATGTGCTTGGCGTCAACGCAGATGGATCAATCAAGGAGTTAACGTACGCCGACATC

TCGCGCCTTCCACTCCTCAATCAAGTTGTCAAGGAGACACTCCGCCTTCATGCTCCCATC

CATTCTATTCTGCGACAAGTCAAGTCTCCGATGCCACTCGAAGGTACACCATACGTTGTC

CCGACCACACACTCCCTCCTTGCTGCACCCGGTGCTACCTCACGAATGGACGAGCACTTC

CCCGAAGCTATGCTGTGGGAACCCCACCGATGGGACGAGAACCCAAGTGAGAAGTACGCA

CATCTCGCACCAAAGCATGTCAAGGAGGGCGTCGCCGAAGAGACTGAAGATTACGGCTAT

GGTCTCGTCAGCAAAGGCGCCGCATCACCATATCTGCCATTCGGTGCTGGCCGACATAGA

TGCATCGGCGAGCAATTCGCCTATGTCCAGCTCCAGACCATCACCTCGGAAGTGATTCGC

GATTTCAAGCTCTACAATGTCGACGGCAGCGACAAAGTTGTCGGCACAGATTACAGTTCG

TTGTTCAGCAGACCTCTCTCGCCAGCCGTCGTGCGANTGGGAGAGGAGAGAAAAGAAATA

GATTTCACAGTAAGA
