# Supplementary material for: A new mechanism for reduced sensitivity to demethylation‐inhibitor fungicides in the fungal banana black Sigatoka pathogen Pseudocercospora fijiensis
Source: Mol Plant Pathol. 2018 Feb 13;19(6):1491–503. doi: 10.1111/mpp.12637 (PMC6637983; doi:10.1111/mpp.12637)
Supplement: Supplementary file 2 — Table S1 Analysis of Pfcyp51 promoter repeats in 225 Pseudocercospora fijiensis isolates from Costa Rica, compared with 14 isolates from other countries. [file MPP-19-1491-s002.docx]

| **Table S1.** Analysis of *Pfcyp51* promoter repeats in 225 *Pseudocercospora* *fijiensis* isolates from Costa Rica, compared with 14 isolates from other countries. | | | | |
| --- | --- | --- | --- | --- |
| No. | Isolate | Origin | Plantation/Location | Repeat units |
| 1 | Ca1.1 | Costa Rica | Cartagena | 2 |
| 2 | Ca1.5 | Costa Rica | Cartagena | 2 |
| 3 | Ca1.7 | Costa Rica | Cartagena | 6 |
| 4 | Ca1.10 | Costa Rica | Cartagena | 6 |
| 5 | Ca1.16 | Costa Rica | Cartagena | 2 |
| 6 | Ca1.20 | Costa Rica | Cartagena | 2 |
| 7 | Ca1.24 | Costa Rica | Cartagena | 6 |
| 8 | Ca2.1 | Costa Rica | Cartagena | 5 |
| 9 | Ca2.5 | Costa Rica | Cartagena | 1 |
| 10 | Ca2.11 | Costa Rica | Cartagena | 1 |
| 11 | Ca2.13 | Costa Rica | Cartagena | 2 |
| 12 | Ca2.15 | Costa Rica | Cartagena | 2 |
| 13 | Ca2.16 | Costa Rica | Cartagena | 2 |
| 14 | Ca2.17 | Costa Rica | Cartagena | 6 |
| 15 | Ca2.19 | Costa Rica | Cartagena | 6 |
| 16 | Ca3.1 | Costa Rica | Cartagena | 2 |
| 17 | Ca3.3 | Costa Rica | Cartagena | 6 |
| 18 | Ca3.5 | Costa Rica | Cartagena | 6 |
| 19 | Ca3.7 | Costa Rica | Cartagena | 6 |
| 20 | Ca3.10 | Costa Rica | Cartagena | 2 |
| 21 | Ca3.14 | Costa Rica | Cartagena | 6 |
| 22 | Ca3.20 | Costa Rica | Cartagena | 2 |
| 23 | Ca3.22 | Costa Rica | Cartagena | 6 |
| 24 | Ca3.24 | Costa Rica | Cartagena | 2 |
| 25 | Ca5.1 | Costa Rica | Cartagena | 6 |
| 26 | Ca5.5 | Costa Rica | Cartagena | 2 |
| 27 | Ca5.7 | Costa Rica | Cartagena | 6 |
| 28 | Ca5.10 | Costa Rica | Cartagena | 2 |
| 29 | Ca5.12 | Costa Rica | Cartagena | 6 |
| 30 | Ca5.13 | Costa Rica | Cartagena | 6 |
| 31 | Ca5.15 | Costa Rica | Cartagena | 6 |
| 32 | Ca5.16 | Costa Rica | Cartagena | 6 |
| 33 | Ca5.17 | Costa Rica | Cartagena | 6 |
| 34 | Ca5.19 | Costa Rica | Cartagena | 6 |
| 35 | Ca6.1 | Costa Rica | Cartagena | 6 |
| 36 | Ca6.3 | Costa Rica | Cartagena | 6 |
| 37 | Ca6.5 | Costa Rica | Cartagena | 6 |
| 38 | Ca6.7 | Costa Rica | Cartagena | 6 |
| 39 | Ca6.9 | Costa Rica | Cartagena | 6 |
| 40 | Ca6.11 | Costa Rica | Cartagena | 6 |
| 41 | Ca6.12 | Costa Rica | Cartagena | 6 |
| 42 | Ca6.15 | Costa Rica | Cartagena | 6 |
| 43 | Ca6.18 | Costa Rica | Cartagena | 6 |
| 44 | Ca7.1 | Costa Rica | Cartagena | 2 |
| 45 | Ca7.5 | Costa Rica | Cartagena | 6 |
| 46 | Ca7.9 | Costa Rica | Cartagena | 2 |
| 47 | Ca7.15 | Costa Rica | Cartagena | 6 |
| 48 | Ca7.18 | Costa Rica | Cartagena | 6 |
| 49 | Ca7.20 | Costa Rica | Cartagena | 2 |
| 50 | Ca7.23 | Costa Rica | Cartagena | 2 |
| 51 | Ca7.28 | Costa Rica | Cartagena | 2 |
| 52 | Ca8.2 | Costa Rica | Cartagena | 6 |
| 53 | Ca8.4 | Costa Rica | Cartagena | 2 |
| 54 | Ca8.8 | Costa Rica | Cartagena | 6 |
| 55 | Ca8.11 | Costa Rica | Cartagena | 6 |
| 56 | Ca8.13 | Costa Rica | Cartagena | 6 |
| 57 | Ca8.16 | Costa Rica | Cartagena | 6 |
| 58 | Ca8.20 | Costa Rica | Cartagena | 6 |
| 59 | Ca8.23 | Costa Rica | Cartagena | 6 |
| 60 | Ca8.26 | Costa Rica | Cartagena | 6 |
| 61 | Ca8.28 | Costa Rica | Cartagena | 2 |
| 62 | Ca8.29 | Costa Rica | Cartagena | 6 |
| 63 | Ca9.1 | Costa Rica | Cartagena | 6 |
| 64 | Ca9.3 | Costa Rica | Cartagena | 2 |
| 65 | Ca9.5 | Costa Rica | Cartagena | 2 |
| 66 | Ca9.8 | Costa Rica | Cartagena | 2 |
| 67 | Ca9.10 | Costa Rica | Cartagena | 2 |
| 68 | Ca9.12 | Costa Rica | Cartagena | 2 |
| 69 | Ca9.14 | Costa Rica | Cartagena | 2 |
| 70 | Ca9.17 | Costa Rica | Cartagena | 2 |
| 71 | Ca9.19 | Costa Rica | Cartagena | 6 |
| 72 | Ca9.22 | Costa Rica | Cartagena | 2 |
| 73 | Ca10.3 | Costa Rica | Cartagena | 6 |
| 74 | Ca10.5 | Costa Rica | Cartagena | 6 |
| 75 | Ca10.7 | Costa Rica | Cartagena | 6 |
| 76 | Ca10.10 | Costa Rica | Cartagena | 1 |
| 77 | Ca10.13 | Costa Rica | Cartagena | 6 |
| 78 | Ca10.23 | Costa Rica | Cartagena | 6 |
| 79 | Ca10.25 | Costa Rica | Cartagena | 6 |
| 80 | Ca10.27 | Costa Rica | Cartagena | 6 |
| 81 | Ca5.18 | Costa Rica | Cartagena | 2 |
| 82 | Ca2.3 | Costa Rica | Cartagena | 6 |
| 83 | SP1.1 | Costa Rica | San Pablo | 3 |
| 84 | SP1.3 | Costa Rica | San Pablo | 1 |
| 85 | SP1.4 | Costa Rica | San Pablo | 3 |
| 86 | SP1.5 | Costa Rica | San Pablo | 3 |
| 87 | SP1.6 | Costa Rica | San Pablo | 2 |
| 88 | SP1.7 | Costa Rica | San Pablo | 1 |
| 89 | SP1.8 | Costa Rica | San Pablo | 3 |
| 90 | SP2.32 | Costa Rica | San Pablo | 6 |
| 91 | SP3.4 | Costa Rica | San Pablo | 3 |
| 92 | SP3.5 | Costa Rica | San Pablo | 3 |
| 93 | SP3.8 | Costa Rica | San Pablo | 2 |
| 94 | SP5.2 | Costa Rica | San Pablo | 6 |
| 95 | SP5.4 | Costa Rica | San Pablo | 6 |
| 96 | SP5.6 | Costa Rica | San Pablo | 6 |
| 97 | SP5.7 | Costa Rica | San Pablo | 3 |
| 98 | SP5.10 | Costa Rica | San Pablo | 3 |
| 99 | SP5.12 | Costa Rica | San Pablo | 3 |
| 100 | SP5.13 | Costa Rica | San Pablo | 3 |
| 101 | SP5.14 | Costa Rica | San Pablo | 6 |
| 102 | SP5.16 | Costa Rica | San Pablo | 6 |
| 103 | SP6.4 | Costa Rica | San Pablo | 3 |
| 104 | SP6.12 | Costa Rica | San Pablo | 3 |
| 105 | SP7.7 | Costa Rica | San Pablo | 1 |
| 106 | SP7.10 | Costa Rica | San Pablo | 3 |
| 107 | SP7.18 | Costa Rica | San Pablo | 1 |
| 108 | SP7.30 | Costa Rica | San Pablo | 1 |
| 109 | SP8.18 | Costa Rica | San Pablo | 3 |
| 110 | SP8.21 | Costa Rica | San Pablo | 6 |
| 111 | SP8.27 | Costa Rica | San Pablo | 6 |
| 112 | SP9.1 | Costa Rica | San Pablo | 3 |
| 113 | SP9.7 | Costa Rica | San Pablo | 1 |
| 114 | SP9.19 | Costa Rica | San Pablo | 3 |
| 115 | SP9.24 | Costa Rica | San Pablo | 3 |
| 116 | SP10.1 | Costa Rica | San Pablo | 1 |
| 117 | SP10.15 | Costa Rica | San Pablo | 1 |
| 118 | SP10.17 | Costa Rica | San Pablo | 3 |
| 119 | SP6.6 | Costa Rica | San Pablo | 3 |
| 120 | SP6.7 | Costa Rica | San Pablo | 3 |
| 121 | SP6.9 | Costa Rica | San Pablo | 3 |
| 122 | SP6.10 | Costa Rica | San Pablo | 3 |
| 123 | SP6.11 | Costa Rica | San Pablo | 3 |
| 124 | SP6.12 | Costa Rica | San Pablo | 6 |
| 125 | SP7.1 | Costa Rica | San Pablo | 6 |
| 126 | Z1.3 | Costa Rica | Zent | 1 |
| 127 | Z.1.5 | Costa Rica | Zent | 1 |
| 128 | Z1.8 | Costa Rica | Zent | 1 |
| 129 | Z1.10 | Costa Rica | Zent | 1 |
| 130 | Z1.12 | Costa Rica | Zent | 1 |
| 131 | Z1.14 | Costa Rica | Zent | 1 |
| 132 | Z1.18 | Costa Rica | Zent | 1 |
| 133 | Z1.20 | Costa Rica | Zent | 1 |
| 134 | Z1.21 | Costa Rica | Zent | 1 |
| 135 | Z1.24 | Costa Rica | Zent | 1 |
| 136 | Z2.1 | Costa Rica | Zent | 1 |
| 137 | Z2.2 | Costa Rica | Zent | 1 |
| 138 | Z2.3 | Costa Rica | Zent | 1 |
| 139 | Z2.5 | Costa Rica | Zent | 1 |
| 140 | Z2.7 | Costa Rica | Zent | 1 |
| 141 | Z2.8 | Costa Rica | Zent | 1 |
| 142 | Z2.9 | Costa Rica | Zent | 1 |
| 143 | Z2.11 | Costa Rica | Zent | 1 |
| 144 | Z2.13 | Costa Rica | Zent | 1 |
| 145 | Z2.14 | Costa Rica | Zent | 1 |
| 146 | Z3.5 | Costa Rica | Zent | 2 |
| 147 | Z3.6 | Costa Rica | Zent | 2 |
| 148 | Z3.11 | Costa Rica | Zent | 2 |
| 149 | Z3.15 | Costa Rica | Zent | 6 |
| 150 | Z3.17 | Costa Rica | Zent | 6 |
| 151 | Z3.32 | Costa Rica | Zent | 2 |
| 152 | Z3.34 | Costa Rica | Zent | 6 |
| 153 | Z4.2 | Costa Rica | Zent | 1 |
| 154 | Z4.7 | Costa Rica | Zent | 1 |
| 155 | Z4.11 | Costa Rica | Zent | 1 |
| 156 | Z4.12 | Costa Rica | Zent | 1 |
| 157 | Z4.14 | Costa Rica | Zent | 1 |
| 158 | Z4.16 | Costa Rica | Zent | 1 |
| 159 | Z4.17 | Costa Rica | Zent | 1 |
| 160 | Z4.19 | Costa Rica | Zent | 1 |
| 161 | Z4.22 | Costa Rica | Zent | 1 |
| 162 | Z4.26 | Costa Rica | Zent | 1 |
| 163 | Z4.29 | Costa Rica | Zent | 1 |
| 164 | Z5.1 | Costa Rica | Zent | 6 |
| 165 | Z5.4 | Costa Rica | Zent | 6 |
| 166 | Z5.6 | Costa Rica | Zent | 6 |
| 167 | Z5.12 | Costa Rica | Zent | 1 |
| 168 | Z5.13 | Costa Rica | Zent | 6 |
| 169 | Z5.15 | Costa Rica | Zent | 6 |
| 170 | Z5.18 | Costa Rica | Zent | 1 |
| 171 | Z5.21 | Costa Rica | Zent | 1 |
| 172 | Z5.25 | Costa Rica | Zent | 1 |
| 173 | Z5.32 | Costa Rica | Zent | 1 |
| 174 | Z6.2 | Costa Rica | Zent | 1 |
| 175 | Z6.3 | Costa Rica | Zent | 1 |
| 176 | Z6.5 | Costa Rica | Zent | 1 |
| 177 | Z6.7 | Costa Rica | Zent | 1 |
| 178 | Z6.9 | Costa Rica | Zent | 1 |
| 179 | Z6.11 | Costa Rica | Zent | 2 |
| 180 | Z6.13 | Costa Rica | Zent | 1 |
| 181 | Z6.15 | Costa Rica | Zent | 2 |
| 182 | Z6.17 | Costa Rica | Zent | 1 |
| 183 | Z7.1 | Costa Rica | Zent | 1 |
| 184 | Z7.7 | Costa Rica | Zent | 6 |
| 185 | Z7.9 | Costa Rica | Zent | 6 |
| 186 | Z7.14 | Costa Rica | Zent | 1 |
| 187 | Z7.18 | Costa Rica | Zent | 6 |
| 188 | Z7.28 | Costa Rica | Zent | 6 |
| 189 | Z7.31 | Costa Rica | Zent | 6 |
| 190 | Z8.1 | Costa Rica | Zent | 2 |
| 191 | Z8.8 | Costa Rica | Zent | 1 |
| 192 | Z8.11 | Costa Rica | Zent | 1 |
| 193 | Z8.12 | Costa Rica | Zent | 2 |
| 194 | Z8.13 | Costa Rica | Zent | 2 |
| 195 | Z8.17 | Costa Rica | Zent | 1 |
| 196 | Z8.18 | Costa Rica | Zent | 2 |
| 197 | Z8.20 | Costa Rica | Zent | 1 |
| 198 | Z8.25 | Costa Rica | Zent | 1 |
| 199 | Z8.27 | Costa Rica | Zent | 2 |
| 200 | Z8.35 | Costa Rica | Zent | 1 |
| 201 | Z10.1 | Costa Rica | Zent | 1 |
| 202 | Z10.3 | Costa Rica | Zent | 1 |
| 203 | Z10.4 | Costa Rica | Zent | 1 |
| 204 | Z10.6 | Costa Rica | Zent | 6 |
| 205 | Z10.7 | Costa Rica | Zent | 1 |
| 206 | Z10.8 | Costa Rica | Zent | 1 |
| 207 | Z10.9 | Costa Rica | Zent | 1 |
| 208 | Z10.10 | Costa Rica | Zent | 1 |
| 209 | Z10.11 | Costa Rica | Zent | 1 |
| 210 | ZTSC2 | Costa Rica | San Carlos | 1 |
| 211 | ZTSC10 | Costa Rica | San Carlos | 1 |
| 212 | ZTSC15 | Costa Rica | San Carlos | 1 |
| 213 | ZTSC40 | Costa Rica | San Carlos | 1 |
| 214 | ZTSC50 | Costa Rica | San Carlos | 1 |
| 215 | ZTSC55 | Costa Rica | San Carlos | 1 |
| 216 | ZTSC60 | Costa Rica | San Carlos | 1 |
| 217 | ZTSC65 | Costa Rica | San Carlos | 1 |
| 218 | ZTSC75 | Costa Rica | San Carlos | 1 |
| 219 | ZTSC80 | Costa Rica | San Carlos | 1 |
| 220 | ZTSC84 | Costa Rica | San Carlos | 1 |
| 221 | ZTSC90 | Costa Rica | San Carlos | 1 |
| 222 | ZTSC95 | Costa Rica | San Carlos | 1 |
| 223 | ZTSC100 | Costa Rica | San Carlos | 1 |
| 224 | ZTSC101 | Costa Rica | San Carlos | 1 |
| 225 | ZTSC77 | Costa Rica | San Carlos | 1 |
| 226 | E22 | Ecuador | a | 1 |
| 227 | GS.4 | Ecuador | a | 1 |
| 228 | GS.10 | Ecuador | a | 1 |
| 229 | RN.3 | Ecuador | a | 1 |
| 230 | RN.5 | Ecuador | a | 1 |
| 231 | RS.13 | Ecuador | a | 1 |
| 232 | SaR.2 | Ecuador | a | 1 |
| 233 | SaR.5 | Ecuador | a | 1 |
| 234 | X845 | Indonesia | a | 1 |
| 235 | X846 | Phillipines | a | 1 |
| 236 | X847 | Taiwan | a | 1 |
| 237 | X849 | Burundi | a | 1 |
| 238 | X851 | Gabon | a | 1 |
| 239 | C86 | Cameroon | a | 1 |
