# Supplementary material for: A new mechanism for reduced sensitivity to demethylation‐inhibitor fungicides in the fungal banana black Sigatoka pathogen Pseudocercospora fijiensis
Source: Mol Plant Pathol. 2018 Feb 13;19(6):1491–503. doi: 10.1111/mpp.12637 (PMC6637983; doi:10.1111/mpp.12637)
Supplement: Supplementary file 3 — Fig. S1 Cross‐resistance between propiconazole and cyproconazole. The 50% inhibitory concentration (EC50) values were determined for both compounds on Pseudocercospora fijiensis colonies for the indicated strains at 10 days post‐inoculation (results are means of three independent experiments). [file MPP-19-1491-s003.docx]

**
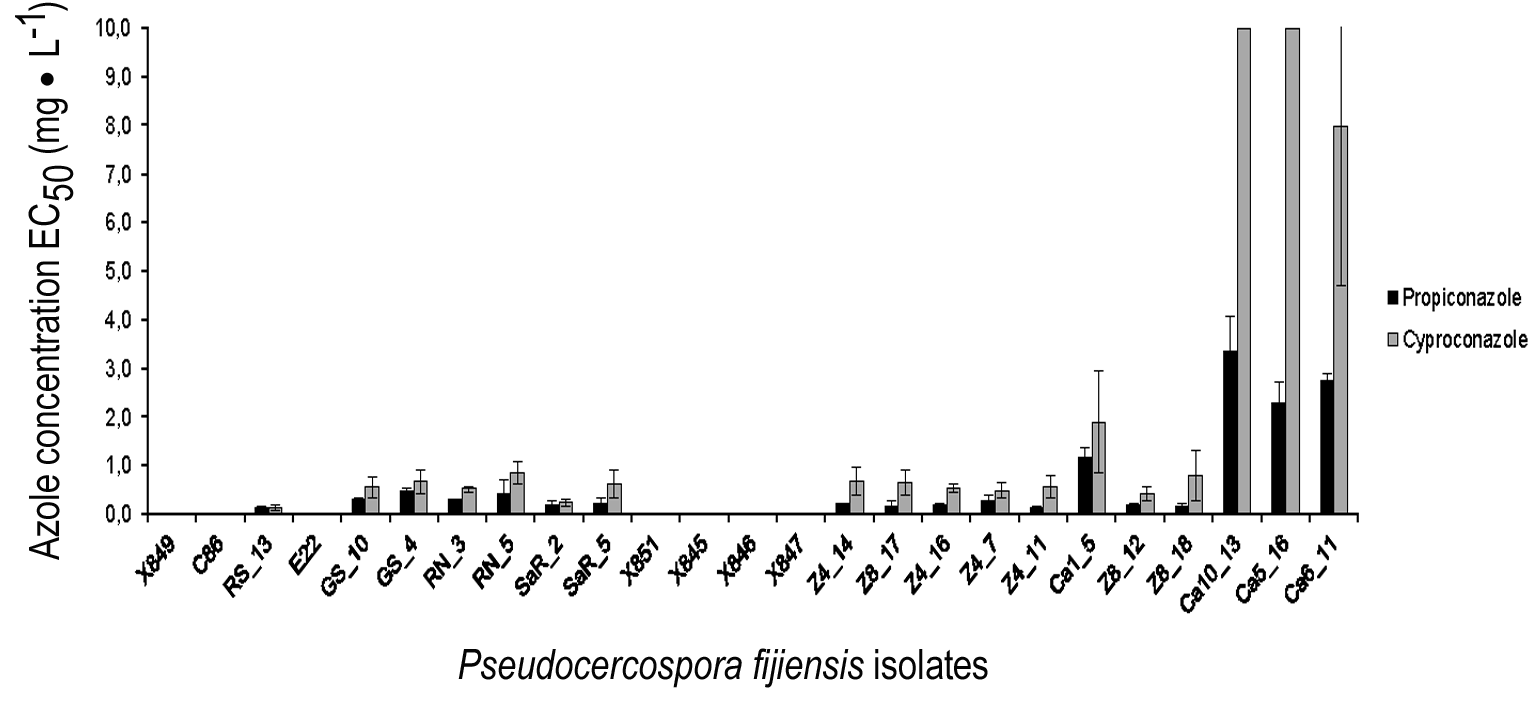
**

**Figure S1.** Cross-resistance between propiconazole and cyproconazole. The EC_50_ values were determined for both compounds on *Pseudocercospora fijiensis* colonies for the indicates strains at 10 days post inoculation (results are means of three independent experiments).
